# Chaperones mainly suppress primary nucleation during formation of functional amyloid required for bacterial biofilm formation†

**DOI:** 10.1039/d1sc05790a

**Published:** 2021-12-13

**Authors:** Madhu Nagaraj, Zahra Najarzadeh, Jonathan Pansieri, Henrik Biverstål, Greta Musteikyte, Vytautas Smirnovas, Steve Matthews, Cecilia Emanuelsson, Janne Johansson, Joel N. Buxbaum, Ludmilla Morozova-Roche, Daniel E. Otzen

**Affiliations:** Interdisciplinary Nanoscience Center (iNANO), Aarhus University Gustav Wieds Vej 14 DK – 8000 Aarhus C Denmark dao@inano.au.dk; Department of Medical Biochemistry and Biophysics, Umeå University 90187 Umeå Sweden; Department of Biosciences and Nutrition, Neo, Karolinska Institutet S – 141 83 Huddinge Sweden; Institute of Biotechnology, Life Sciences Center, Vilnius University Vilnius Lithuania; Department of Life Sciences, Imperial College London, South Kensington Campus London SW72AZ UK; Department of Biochemistry and Structural Biology, Center for Molecular Protein Science, Lund University PO Box 124 SE-22100 Lund Sweden; The Scripps Research Institute 10550 North Torrey Pines Road La Jolla CA 92037 USA

## Abstract

Unlike misfolding in neurodegenerative diseases, aggregation of functional amyloids involved in bacterial biofilm, *e.g.* CsgA (*E. coli*) and FapC (*Pseudomonas*), is carefully regulated. However, it is unclear whether functional aggregation is inhibited by chaperones targeting pathological misfolding and if so by what mechanism. Here we analyze how four entirely different human chaperones or protein modulators (transthyretin, S100A9, Bri2 BRICHOS and DNAJB6) and bacterial CsgC affect CsgA and FapC fibrillation. CsgA is more susceptible to inhibition than FapC and the chaperones vary considerably in the efficiency of their inhibition. However, mechanistic analysis reveals that all predominantly target primary nucleation rather than elongation or secondary nucleation, while stoichiometric considerations suggest that DNAJB6 and CsgC target nuclei rather than monomers. Inhibition efficiency broadly scales with the chaperones' affinity for monomeric CsgA and FapC. The chaperones tend to target the most aggregation-prone regions of CsgA, but do not display such tendencies towards the more complex FapC sequence. Importantly, the most efficient inhibitors (Bri2 BRICHOS and DNAJB6) significantly reduce bacterial biofilm formation. This commonality of chaperone action may reflect the simplicity of functional amyloid formation, driven largely by primary nucleation, as well as the ability of non-bacterial chaperones to deploy their proteostatic capacities across biological kingdoms.

## Abstract 2

Unlike misfolding in neurodegenerative diseases, aggregation of functional amyloids involved in bacterial biofilm, *e.g.* CsgA (*E. coli*) and FapC (*Pseudomonas*), is carefully regulated.

## Graphical abstract

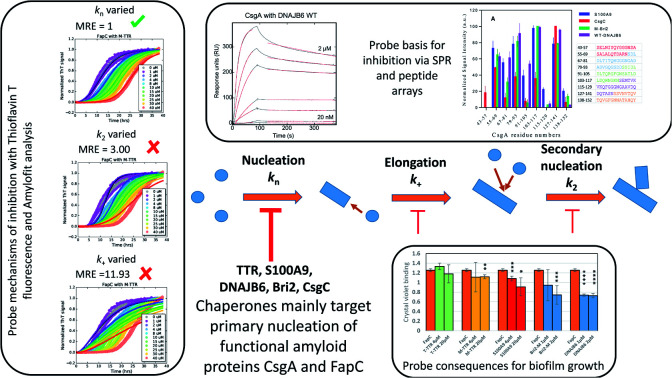

## Introduction

Amyloid deposits formed as a result of the aggregation of misfolded proteins are associated with a wide range of human systemic and neurodegenerative diseases.^1,2^ While we still struggle to achieve a detailed understanding of the mechanistic details of amyloid biogenesis and the resulting pathology, the last decade has seen enormous advances utilizing interdisciplinary approaches and newer techniques available to attack the problem.^3,4^ It is now clear that while all amyloids share a defining set of biophysical characteristics, not all are associated with human or animal disease states. Functional amyloids are found in many different species throughout nature. Their biological roles vary widely. Some incorporate the pigment melanin into melanosomes for UV protection,^5^ while others constitute reservoirs for peptide hormones,^6^ act as information carriers^7^ or are found on the surface of bacteria and fungi as structural components.^8,9^ For example, CsgA in *E. coli* and FapC in *Pseudomonas* form amyloid fibrils extending from the bacterial surface which mechanically strengthen the biofilm,^10,11^ increase cell hydrophobicity^12^ and promote binding to eukaryotic host cells through direct contact with fibronectin.^13^ These phenomena increase bacterial resistance to antibiotic treatment of infections in humans. Despite their biological diversity, all amyloid fibrils (functional or pathologic) share the characteristic cross-β architecture,^14^ diminished solubility under physiological conditions of pH and ionic strength, binding of the dye Congo red with birefringence when examined under polarized light and the ability to bind and increase fluorescence of Thioflavin T.^15^ This raises the question whether they also display similar sensitivity to inhibition of aggregation by *e.g.* chaperones.

Maintaining protein homeostasis poses a formidable challenge for the cell and, on the metazoan scale, to the organism. To this end, Nature has employed a versatile collection of intra- and extracellular molecular chaperones to control protein quality, preventing or reversing misfolding and eliminating potentially pathogenic aggregates to reduce cell and tissue toxicity.^16–19^ In misfolding diseases, excessive misfolding or unfolding eventually overwhelms the chaperone system and allows aggregates to accumulate.^20,21^ In contrast, the production of functional amyloids in bacteria (FuBA) usually involves the co-expression of dedicated helper proteins to prevent unwanted aggregation inside the cell and assist in transporting the amyloid protein to the cell surface to form biofilms.^22,23^ Both CsgA and FapC are shunted through the outer membrane *via* a dedicated membrane channel (CsgG^24^ and FapF,^22^ respectively). Fibrillation on the bacterial surface is initiated by a nucleator protein (CsgB^25^ and – most likely – FapB^26^). CsgA is maintained in an unfolded state in the periplasm prior to export through dynamic and electrostatically driven^27^ transient binding to the chaperone CsgC,^22^ which can maintain CsgA in an unfolded state at CsgC : CsgA mole ratios as low as 1 : 40.^28^ CsgC also inhibits the aggregation of the pathological amyloid α-synuclein (αSN) through a shared sequence motif.^22^

Our study addresses the question of whether a set of human proteins, known to inhibit pathological amyloid formation *in vitro* and in a variety of physiological settings, will have a similar effect on functional amyloids. Such insight can extend our understanding of ways to combat pathological and functional amyloidogenesis, potentially advancing therapeutic strategies and allowing control of undesirable biofilm formation in a variety of environments.^29^ We investigate four different human proteins (transthyretin (TTR), S100A9, Bri2 BRICHOS, DNABJ6), together with the known bacterial chaperone CsgC, for their ability to inhibit fibrillation of FuBA. For simplicity we refer to all five proteins as chaperones, although only DNABJ6 and CsgC are conventionally classified in this way. Our aim is to determine if these molecules show individual modes of action or share common mechanistic features. As FuBA we use CsgA and FapC.

### TTR

The human systemic amyloid precursor protein transthyretin (TTR) is a homotetramer, which inhibits Aβ fibrillation by arresting primary and surface catalysed secondary nucleation. This may account for TTR's ability to suppress neuropathological and behavioral manifestations in transgenic models of human Alzheimer's disease (AD).^30–33^*In vitro* TTR inhibits Aβ aggregation by two distinct mechanisms. TTR tetramers (T-TTR) bind Aβ monomers as well as oligomers and fibrils, while an engineered monomeric TTR variant (M-TTR, containing a double mutation F87M/L110M which prevents it from forming native tetramers through the introduction of steric clashes^34^) forms oligomers which interact with Aβ oligomers, inhibiting fibrillogenesis but allowing the formation of large amorphous, non-cytotoxic aggregates.^35^ Like Bri2 (*vide infra*), it inhibits IAPP cytotoxicity *in vitro*. Relative to M-TTR, T-TTR showed a modest (10%) ability to inhibit CsgA fibrillation.^36^ A 2 : 1 M-TTR : CsgA molar ratio led to a 4-fold increase in the half-time of fibrillation.^36^ It remains unclear whether M-TTR's enhanced inhibition is related to oligomer–oligomer interactions, as seen in its interaction with Aβ, or its structural similarity to CsgC.^36^ TTR does not affect non-amyloidogenic protein aggregation (West *et al.*, submitted).

### S100A9

S100A9 is a specific pro-inflammatory mediator implicated in neurodegenerative diseases, including Parkinson's (PD) and AD. A homolog of calcium-binding S100 protein, S100A9 colocalises and co-aggregates with αSN^37^ within neuronal inclusions known as Lewy bodies, which represent hallmarks of PD development. S100A9 is fibrillogenic *in vitro* and accumulates as *Corpora amylacea* inclusions in the prostate where they are associated with inflammation and possible malignancy.^38^ Co-incubation of αSN and S100A9 leads to faster aggregation but the formation of less toxic oligomers than those formed by S100A9 alone (although the hybrids are just as toxic as pure αSN oligomers).^37^ S100A9 also co-aggregates with Aβ, triggering a neuroinflammatory amyloid cascade, which leads to Aβ-containing senile plaque formation in AD.^39^ The latter two instances may involve cross-seeding.

### Bri2

The Bri2 protein, associated with Familial British and Danish dementias, comprises a β-rich BRICHOS domain^40^ which inhibits secondary nucleation and elongation during Aβ fibrillization,^41^ reducing Aβ neurotoxicity in a *Drosophila* model.^42^ Recombinant Bri2-BRICHOS (henceforth referred to as Bri2 in this study) forms both monomers, dimers and oligomers. Commensurate with their sizes, monomers (M-Bri2) bind monomeric Aβ and suppress neurotoxicity, dimers inhibit fibrillar aggregation and oligomers (O-Bri2) suppress unspecific (non-fibrillar) aggregation brought about by *e.g.* thermal denaturation.^43^ In addition, Bri2 inhibits fibrillation and toxicity of the IAPP peptide whose aggregation is associated with type 2 diabetes.^44^

### DNAJB6

The human molecular chaperone DNAJB6 is a member of the Hsp40 small heat shock protein family and reduces aggregation of polyQ peptides^45^ and Aβ42 (ref. 46) by inhibiting primary nucleation.^41^ This involves binding to Aβ42 oligomers *via* the functionally important S/T region rich in Ser/Thr residues.^47^

Thus TTR, Bri2 BRICHOS and DNAJB6 differ in their mechanisms of inhibition of aggregation of pathological proteins, whereas S100A9 can be seen more as a promoter of aggregation.^48,49^ We have studied the kinetics of aggregation of CsgA and FapC in the presence of increasing concentrations of these chaperones. Normalized fibrillation time courses are analyzed using the programme Amylofit^50^ which allows us to determine the mechanism of aggregation and define which microscopic aggregation step is inhibited by the chaperones. Both CsgA and FapC predominantly aggregate *via* a “fast track” pathway involving primary nucleation and elongation,^51^ although fragmentation can make a significant contribution *e.g.* when FapC is destabilized by the removal of one or more of its three imperfect repeats.^52^ Strikingly, all five chaperones mainly target primary nucleation and elongation, indicating a preference for binding to the monomer species as well as the growing ends of the fibril. CsgA is more sensitive to chaperone inhibition than FapC, possibly because of its minimalist amyloid-repeat structure. Importantly, inhibitory effects are also observed *in vivo* using biofilm assays. Thus, all chaperones appear to target similar features in the aggregation process, namely the monomer or nucleus of fibrillation, indicating a commonality in their mode of action. This in turn suggests a strategy to target functional microbial aggregates *via* the monomeric or nucleated state.

## Results

### M-TTR but not T-TTR strongly inhibits fibrillation of CsgA and FapC

**Fig. 1:**
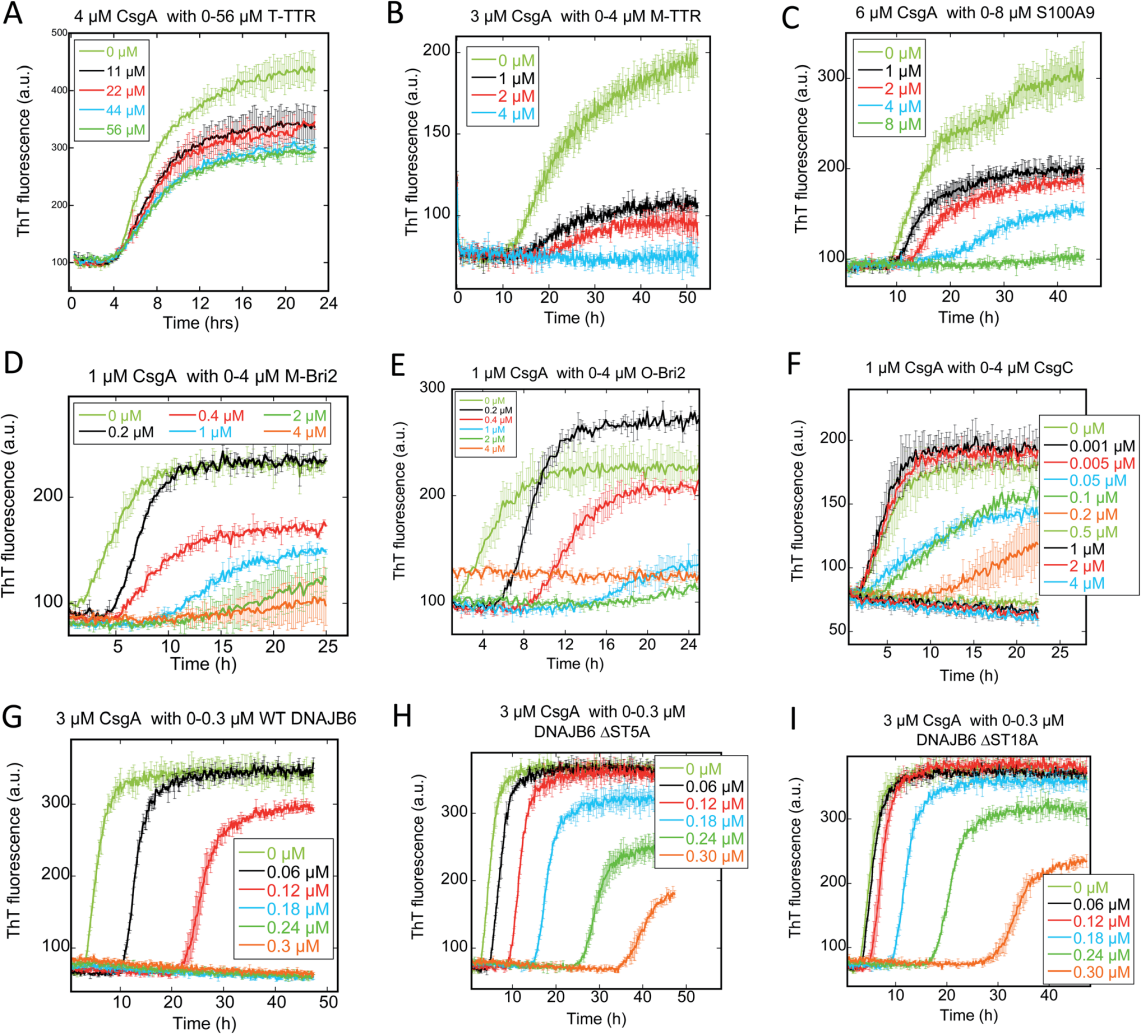
Raw ThT fluorescence time curves depicting the fibrillation of CsgA in the presence of nine different chaperones (A) TTR, (B) M-TTR, (C) S100A9, (D) monomeric-Bri2, (E) oligomeric-Bri2, (F) CsgC, (G) DNAJB6 WT, (H) DNAJB6 ΔST5A, (I) DNAJB6 ΔST18A. Concentrations of both CsgA and chaperones are indicated in each panel. The aggregation kinetics was measured by ThT fluorescence every 10 min at 25 °C under shaking conditions.

**Fig. 2:**
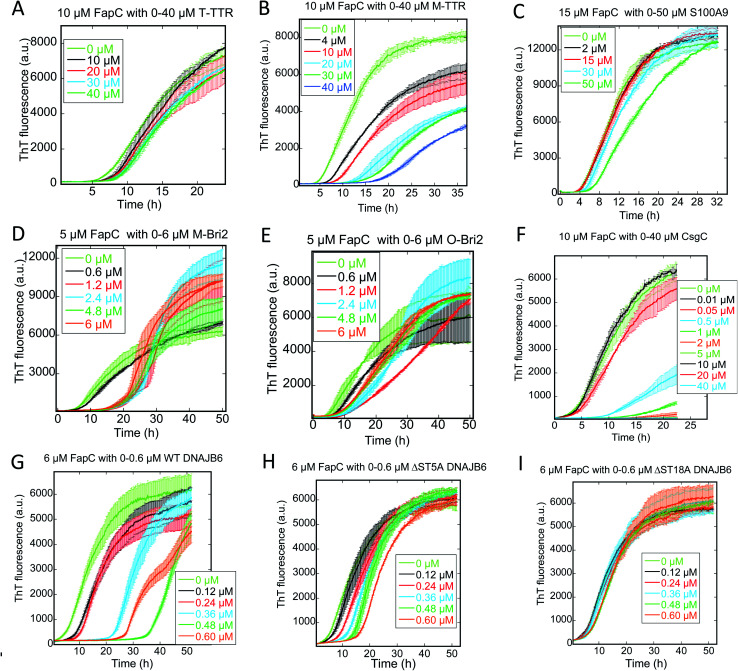
Raw ThT fluorescence time curves depicting the fibrillation of FapC in the presence of nine different chaperones (A) TTR, (B) M-TTR, (C) S100A9, (D) monomeric-Bri2, (E) oligomeric-Bri2, (F) CsgC, (G) DNAJB6 WT, (H) DNAJB6 ΔST5A, (I) DNAJB6 ΔST18A. Concentrations of both FapC and chaperones are indicated in each panel. The aggregation kinetics was as measured by ThT fluorescence every 10 min at 25 °C under shaking conditions.

We followed the kinetics of aggregation of the functional amyloids CsgA and FapC in the presence of increasing concentrations of the different chaperones. Aggregation was monitored using the dye ThT, whose intrinsic fluorescence increases dramatically upon binding to fibrils.^15^ All runs are recorded in triplicates and the average run is shown with error bars. A typical fibrillation curve is sigmoidal and consists of a largely flat lag phase, followed by a steep growth phase that eventually leads to a plateau. Generally, very little variation in lag time was observed between triplicate runs. ThT time curves for FapC and CsgA alone and with chaperones are presented in Fig. 1 and 2 in a sequence matching their description in the main text. On their own, both CsgA and FapC show a visible lag phase, which is typical of a nucleation–elongation mechanism.

Consistent with previous reports,^36^ T-TTR has only a modest effect on CsgA fibrillation, slightly reducing end point ThT levels without altering the lag phase (Fig. 1A). In contrast, M-TTR completely suppresses CsgA fibril formation at M-TTR : CsgA molar ratios at 1 and above (Fig. 1B). Remarkably, M-TTR (but not T-TTR) also reduces fibrillation of FapC (Fig. 2A and B), although the effect is not as pronounced as its impact on CsgA. Even at a molar ratio of 4 : 1 M-TTR : FapC, aggregation is delayed but not entirely abolished.

### CS100A9 inhibits CsgA fibrillation to a much greater extent than FapC fibrillation

A similar inhibition pattern was seen for the pro-inflammatory mediator S100A9, which efficiently inhibited CsgA aggregation in a concentration-dependent manner with essentially complete inhibition at molar S100A9 : CsgA ratios > 1 (Fig. 1C). In contrast, FapC fibrillation was only modestly slowed by S100A9 (Fig. 2C).

**Fig. 3:**
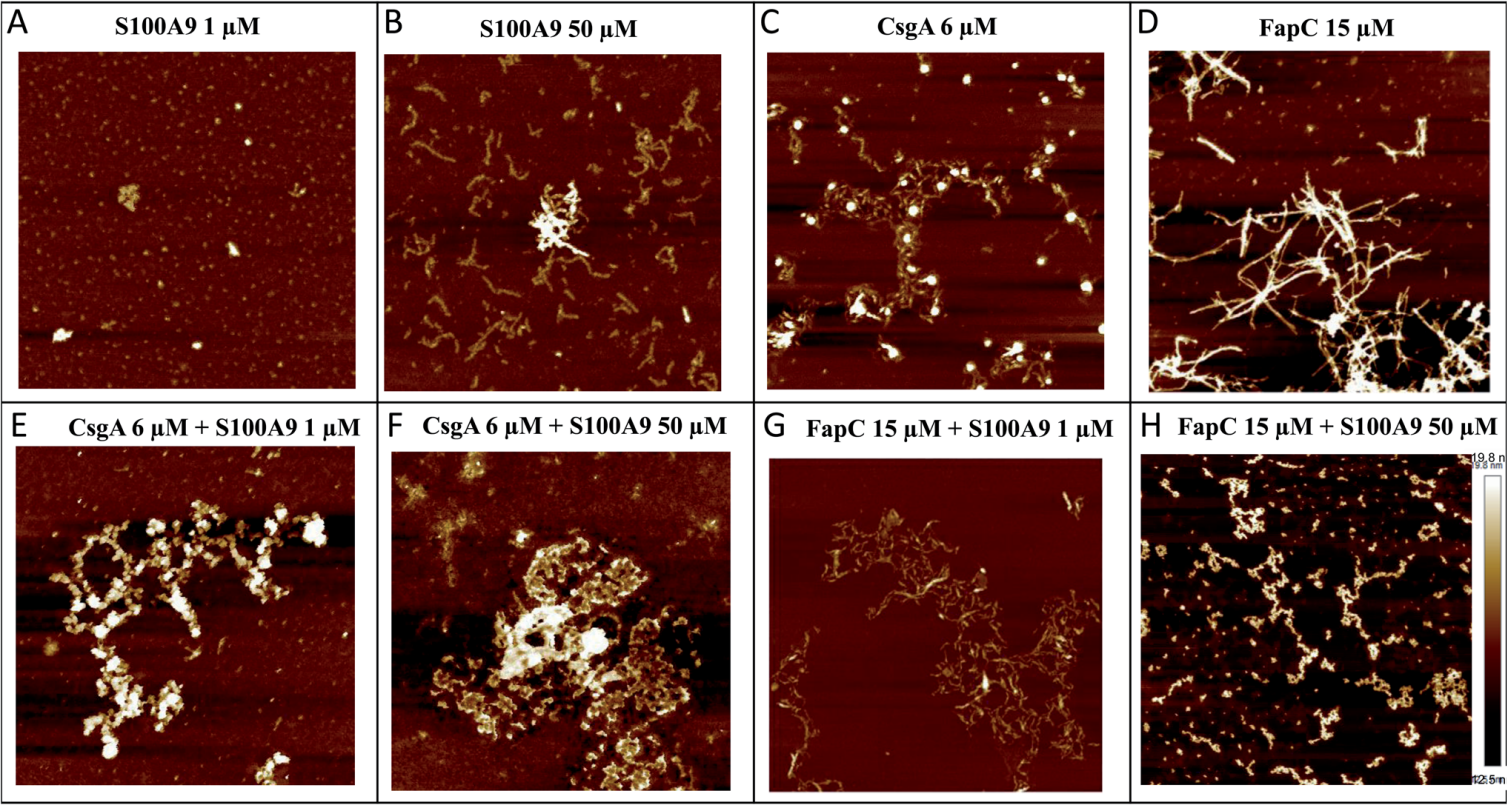
Architecture of aggregates of CsgA and FapC alone and in the presence of S100A9. The end point of the aggregation kinetics were used for AFM analysis (A), (B) S100A9 alone 1–50 μM, (C) CsgA alone 6 μM, (D) FapC alone 15 μM, (E) CsgA 6 μM co-aggregated with S100A9 1 μM, (F) CsgA 6 μM co-aggregated with S100A9 50 μM, (G) FapC 15 μM co-aggregated with S100A9 1 μM, (H) FapC 15 μM co-aggregated with S100A9 50 μM Images obtained by AFM. Height scale bar provided in panel H.

We used AFM to analyze the structures of the FuBA aggregates formed after 35–40 h of incubation in the presence of S100A9 (Fig. 3A–H). S100A9 aggregates formed on their own are highly dependent on S100A9 concentration. At 1 μM, only small spherical structures are observed within the incubation period (Fig. 3A), but at 50 μM the protein forms thin (2 nm in height) and short coiled fibrils (Fig. 3B). CsgA (Fig. 3C) and FapC (Fig. 3D) form longer (several μm) fibrils (8–10 nm in height) which entangle to a mesh or network. When co-incubated, S100A9 and CsgA formed large clumps of amorphous aggregates (Fig. 3E and F), demonstrating how S100A9 diverts aggregation away from fibrillation towards more disorganized self-association. This is very similar to previous reports of M-TTR interactions with Aβ,^53^ HypF-N^54^ and CsgA.^36^ Fibrils were still formed when FapC was co-aggregated with S100A9, confirming S100A9's retardation but not complete suppression of FapC's ThT fluorescence signals (Fig. 3G and H). Also there were changes to the general appearance of the fibrils towards thinner, shorter and more curly fibrils and clumpy aggregates.

### Bri2 monomer and oligomer both inhibit FuBA fibrillation

Both monomeric (M-Bri2, Fig. 1D) and oligomeric (O-Bri2, Fig. 1E) Bri2 inhibited CsgA aggregation in a concentration dependent manner, leading to near-complete suppression at equimolar concentrations. There was very little difference between the performance of M-Bri2 and O-Bri2, unlike their effect on Aβ aggregation, where M-Bri2 was twice as efficient as O-Bri2 on a monomer concentration basis.^43^

**Fig. 4:**
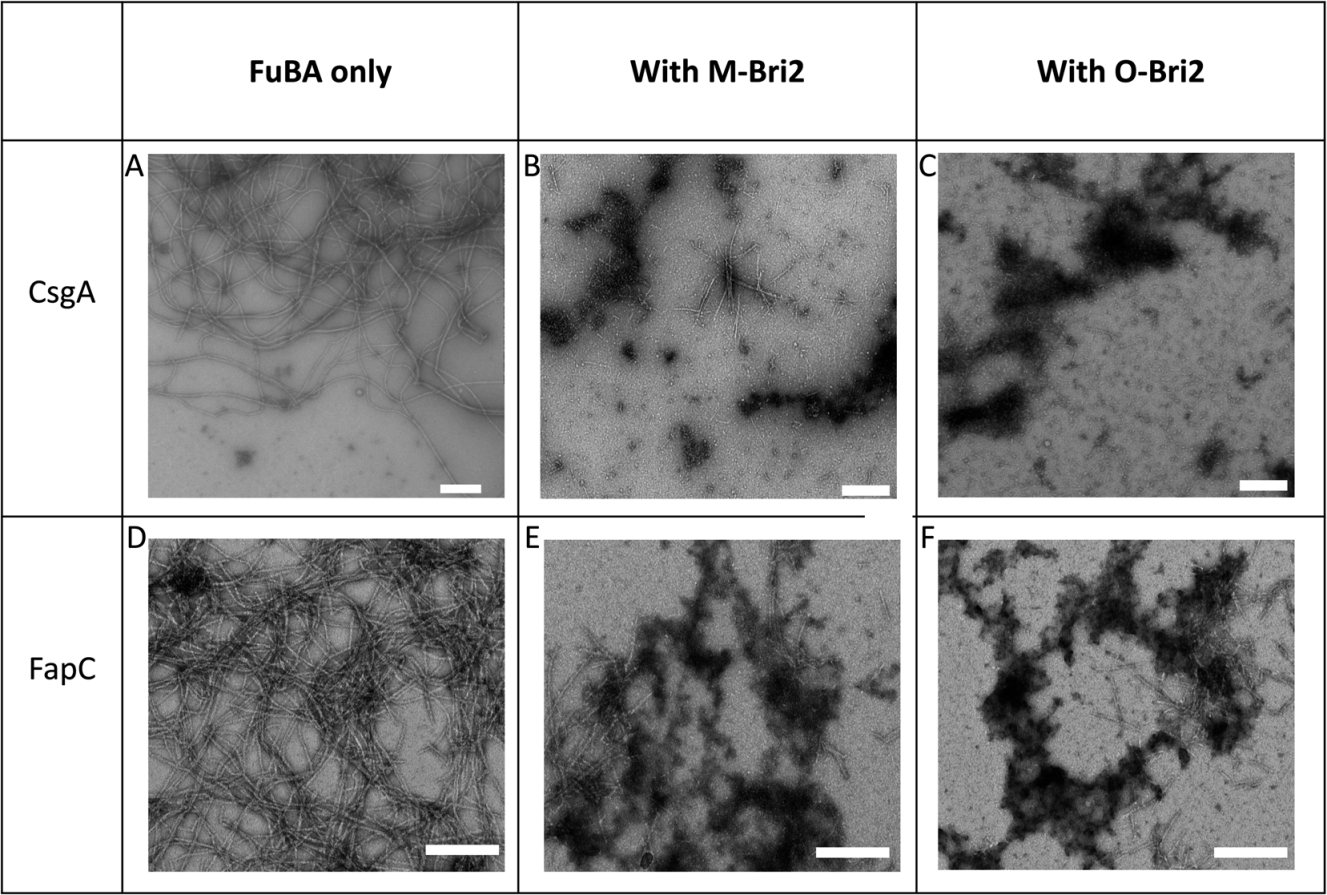
Architecture of aggregates of 1 μM CsgA (A), (B) CsgA 6 μM in the presence of Bri2 monomer (M-Bri2) and (C) with oligomer (O-Bri2). FapC (D) alone, (E) FapC in the presence of Bri2 monomer (M-Bri2) and (F) with oligomer (O-Bri2). Chaperone concentrations were 4 μM with CsgA and 6 μM with FapC. Images obtained by Transmission Electron Microscopy. Scale bar indicates 200 nm.

M-Bri2 (Fig. 2D) and O-Bri2 (Fig. 2E) did not inhibit FapC aggregation as much as CsgA, but only modestly slowed down fibrillation. According to electron microscopy, CsgA (Fig. 4A) and FapC (Fig. 4D) formed thin and undecorated fibrils on their own. However, both M-Bri2 and O-Bri2 led to dense clumps of amorphous aggregates around the fibrils formed by CsgA (Fig. 4B and C) and FapC (Fig. 4E and F), with FapC showing a greater tendency to form fibrils along with amorphous aggregates.

### DNAJB6 inhibits FuBA fibrillation at sub-stoichiometric concentrations and is dependent on a conserved S/T-rich region

**Fig. 5:**
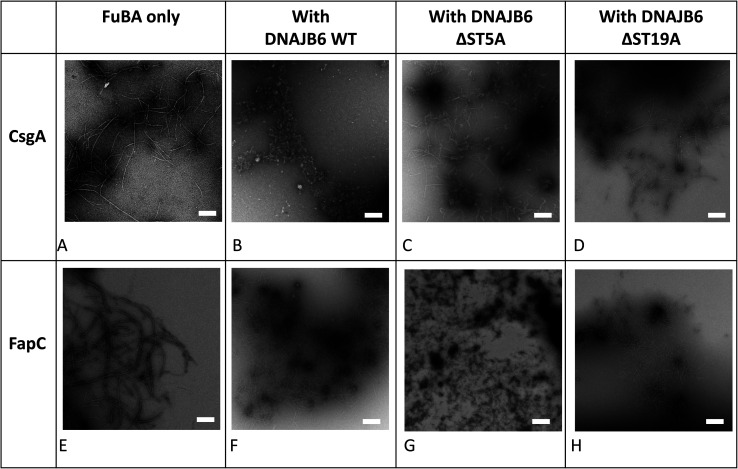
Architecture of aggregates of 3 μM CsgA (A), (B) CsgA in the presence of DNAJB6 WT and the truncation mutants ΔST5A (C) and ΔST18A (D). 6 μM FapC alone (E), (F) FapC in the presence of DNAJB6 WT and the truncation mutants ΔST5A (G) and ΔST18A (H). Chaperone concentrations were 0.3 μM with CsgA and 0.6 μM with FapC. Images obtained by Transmission Electron Microscopy. Scale bar indicates 200 nm.

The inhibitory activity of the heat shock protein DNABJ6 is dependent on the S/T rich region, which is located largely within the disordered middle domain. Accordingly, we also included the partially inactivated variant ΔST5A (in which only 5 Ser/Thr residues have been mutated) and the inactive mutant ΔST18A (in which 18 Ser and Thr residues are mutated to Ala, leading to complete loss of function^55^). Remarkably, WT DNABJ6 turned out to be the most potent aggregation inhibitor on a molar basis. As little as 2% WT DNABJ6 doubled the half time of fibrillation, and 6% completely inhibited fibrillation (Fig. 1G). A lower but still significant degree of sub-stoichiometric inhibition was seen for FapC (Fig. 2G). A diminishing effect was seen for ΔST5A and ΔST18A for both CsgA (Fig. 1H and I) and FapC (Fig. 2H and I) (particularly pronounced for FapC), consistent with their decreasing inhibitory effects towards Aβ aggregation.^55^ EM images showed replacement of the straight long fibrils formed by FuBA alone (Fig. 5A and E) with short worm-like fragments in the presence of WT DNABJ6 (Fig. 5B and F) and fibrils of increasing length (but also decorated with amorphous structures) in the presence of the two variants with reduced activity (Fig. 5C, D, G and H).

### CgsC inhibits fibrillation of both CsgA and FapC, but less efficiently than DNAJB6

Consistent with its biological role as chaperone for CsgA, the protein CsgC showed a pronounced ability to suppress CsgA aggregation, leading to complete inhibition of aggregation at 1 : 2 CsgC : CsgA mole ratios (Fig. 1F). However, this was less efficient than DNAJB6 which achieved complete suppression at 0.18 : 3 (*i.e.* 0.12 : 2) mole ratio. Nevertheless, CsgC also suppressed aggregation of FapC (despite its lack of homology with CsgA^56^) and with comparable efficiency, *i.e.* complete cessation of fibrillation at 1 : 2 CsgC : FapC molar ratios (Fig. 2F).

### Kinetic analysis of unseeded and seeded reactions reveals that chaperones mainly suppress primary nucleation but may also affect secondary nucleation

**Fig. 6:**
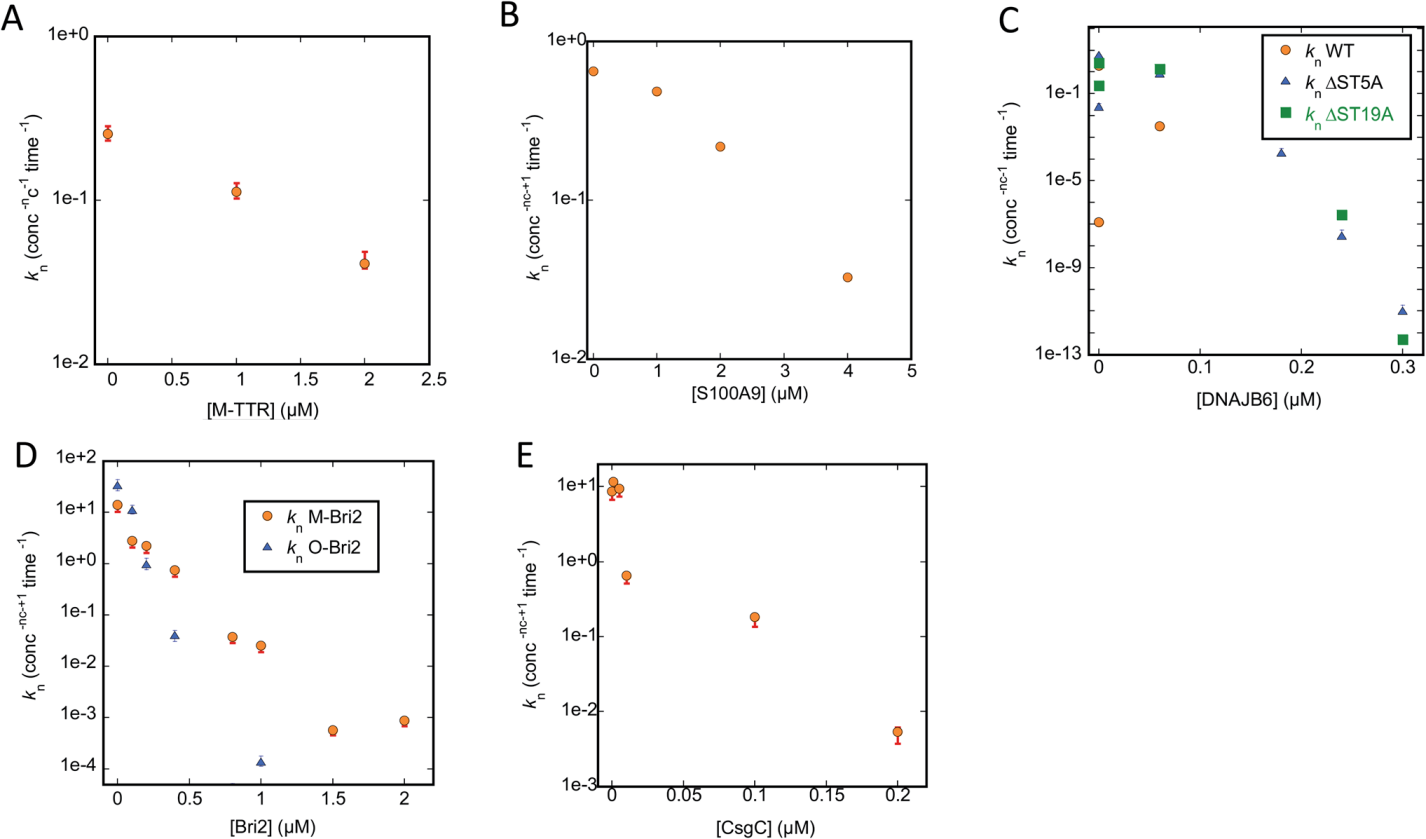
Primary nucleation rate constant *k*_n_ of CsgA aggregation plotted against chaperone concentration. Values for *k*_n_ obtained from the best fit obtained by varying only *k*_n_ for the CsgA aggregation curves in the presence of different chaperones (raw ThT curves presented in Fig. S1–S3†). Chaperones involved were (A) M-TTR, (B) S100A9, (C) DNAJB6 WT and the truncation mutants ΔST5A and ΔST18A, (D) monomeric-Bri2 and oligomeric-Bri2 and (E) CsgC.

To analyse our kinetic data in a systematic fashion that allows us to compare the effects of different chaperones, we turn to Amylofit. This web-server programme^50^ performs global fits on normalized ThT-based time curves of aggregation (in our case simultaneous fitting of multiple time curves recorded at different concentrations of chaperone) to evaluate which aggregation mechanism(s) best describes the experimental data. Time curves in which the signal does not change due to complete suppression of aggregation have to be excluded, which limits the analysis to chaperone conditions in which a sigmoid curve is observed. We fit our data using an aggregation model which includes both primary nucleation (formation of a nucleus from monomers), elongation (extension of the two growing ends of each fibril) and secondary nucleation (formation of nuclei along the side of the fibrils). We also tried to include fragmentation (breakage of existing fibrils, leading to shorter fibrils and a larger number of growing ends), but this led to worse fits (data not shown) and is therefore not included in our results. Our strategy was to carry out a global fit of ThT time curves collected at different chaperone concentrations, in which we allow only one rate constant to vary within the model, either nucleation (*k*_n_), elongation (*k*_+_) or secondary nucleation (*k*_2_). This allowed us to determine whether the chaperone targets one specific step in the aggregation mechanism or several. For all data sets analysed using different models, we provide the Mean Squared Residual Error (MRE), normalized to the value provided by the model with the lowest MRE value. Based on these values and the visible quality of the fits, we indicate the best models for the chaperone-FuBA system in question. In essentially all cases, variations in *k*_n_ turns out to be the best way to account for the data. The primary nucleation constant from the best fits and corresponding plots of (log)*k*_n_*versus* [chaperone] are shown in Fig. 6A–E (CsgA) and Fig. 8A–E (FapC); all fits based on variation of *k*_n_, *k*_+_ and *k*_2_ are shown in ESI Fig. S1–S3 for CsgA and Fig. S4–S6† for FapC.

We start by describing the results of fitting our data to aggregation of CsgA (Fig. S1–S3†). For T-TTR, the normalized ThT time curves recorded at different T-TTR concentrations overlap almost completely, precluding meaningful analysis (Fig. S1A, D and G†). There is a slightly greater effect in the case of M-TTR, but the variation is so small that, although the kinetic data can be fitted satisfactorily, there is no significant difference in quality when varying either *k*_n_, *k*_2_ or *k*_+_ (Fig. S1B, E and H†). All rate constants decline in a log-linear fashion within the concentration range that provides sigmoidal curves, though *k*_n_ does decline by a factor of 6 (Fig. 6A) while the two other constants only decline ∼1.5 fold (data not shown).

S100A9 shows a stronger impact on ThT curves than TTR, but again it is not meaningful to distinguish between different scenarios, since Amylofit can fit the data almost equally well by varying either *k*_n_, *k*_2_ or *k*_+_ (Fig. S1C, F and I†). Nevertheless, again *k*_n_ varies more (*ca.* 20-fold, Fig. 6B) than the other rate constants (3–7-fold, data not shown).

However, the situation becomes clearer when we turn to DNAJB6, the most efficient chaperone (Fig. S2†). Both for WT (Fig. S2A, D and G†) and the increasingly attenuated variants ΔST5A (Fig. S2B, E and H†) and ΔST18A (Fig. S2C, F and I†), variation of *k*_n_ fits the data visibly better than varying *k*_2_ or *k*_+_; furthermore, the value of *k*_n_ declines more steeply with [chaperone] for the wildtype than for the two attenuated mutants (Fig. 6C).

The superior performance of *k*_n_ in fitting data is also demonstrated when we turn to M-Bri2 (Fig. S3A, D and G†) and O-Bri2 (Fig. S3B, E and H†). Although the relative MRE values do not vary much from model to model (due to the high noise levels at high chaperone concentrations arising from the low growth in ThT signal), the fits are of markedly better quality when *k*_n_ is varied. O-Bri2 leads to a steeper decline in *k*_n_ than M-Bri2 (Fig. 6D; note that concentration is given in units of M-Bri2).

With CsgC (Fig. S3C, F and I†), variation of *k*_n_ leads to the best fit to the kinetic data and a 1000-fold variation in values between 0 and 0.2 μM CsgC (Fig. 6E), though MRE values (and fit qualities) are only slightly inferior for *k*_2_ and *k*_+_. There was also a tendency for *k*_+_ to lead to slightly better fits than *k*_2_.

Since Amylofit also allows us to vary compound parameters such as the product *k*_n_*k*_+_, we compared the fit quality for models in which either *k*_n_ or *k*_n_*k*_+_ were varied. In all cases except 3, the MRE value was unaltered; amongst these 3 cases, variation in *k*_n_*k*_+_ improved MRE by 33% for one chaperone (CsgA with Bri-O) but made it worse by 28 and 71% for two others (CsgA with DNAJB6Δ5ST and S100A9). Therefore we conclude that changes in *k*_n_ (Fig. 6A–E) are sufficient to explain the impact of the chaperones on CsgA aggregation, though changes in secondary nucleation and elongation may play a minor part.

**Fig. 7:**
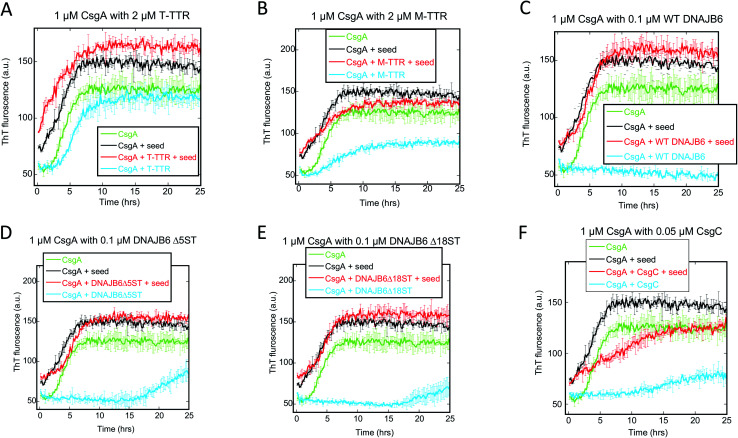
Seeding experiments with 1 μM CsgA using 50 nM CsgA seeds (in monomer units) in the presence or absence of the indicated concentrations of different chaperones. At these chaperone concentrations, there is significant inhibition of aggregation, but not complete repression (Fig. 1). The chaperones were (A) T-TTR, (B) M-TTR (C) DNAJB6 WT, (D) DNAJB6 ΔST5A (E) DNAJB6 ΔST18A and (F) CsgC. Controls include CsgA alone, CsgA in the presence of chaperone without fibrils and CsgA in presence of seeds/fibrils without chaperones.

Given that primary nucleation rather than elongation or secondary nucleation appears to be the main target for these chaperones, we sought to confirm this conclusion using seeding experiments for a selection of chaperones, data for which are shown in Fig. 7A–F. Addition of pre-formed fibrils bypasses the need for new nuclei (*i.e.* primary nucleation) and thus diminishes the effect of chaperones unless the chaperone also target elongation and secondary nucleation. In the absence of chaperones, CsgA fibrillation is accelerated by the addition of seeds, and 5% seed (w/w) eliminates the lag phase. Neither T-TTR nor M-TTR change this acceleration. In contrast to prior observations showing the inhibition of CsgA by both forms of TTR (M-TTR > T-TTR),^36^ our studies showed little effect of these two chaperones on CsgA fibrillation (Fig. 7A and B). It is noteworthy that, in our hands, the otherwise highly potent aggregation inhibitor DNAJB6 and its variants have no effect on aggregation of seeded CsgA either (Fig. 7C–E) and CsgC has only a very modest effect (Fig. 7F). This is consistent with our previous conclusion that these chaperones target the nucleation step, which is eliminated through seeding.

**Fig. 8:**
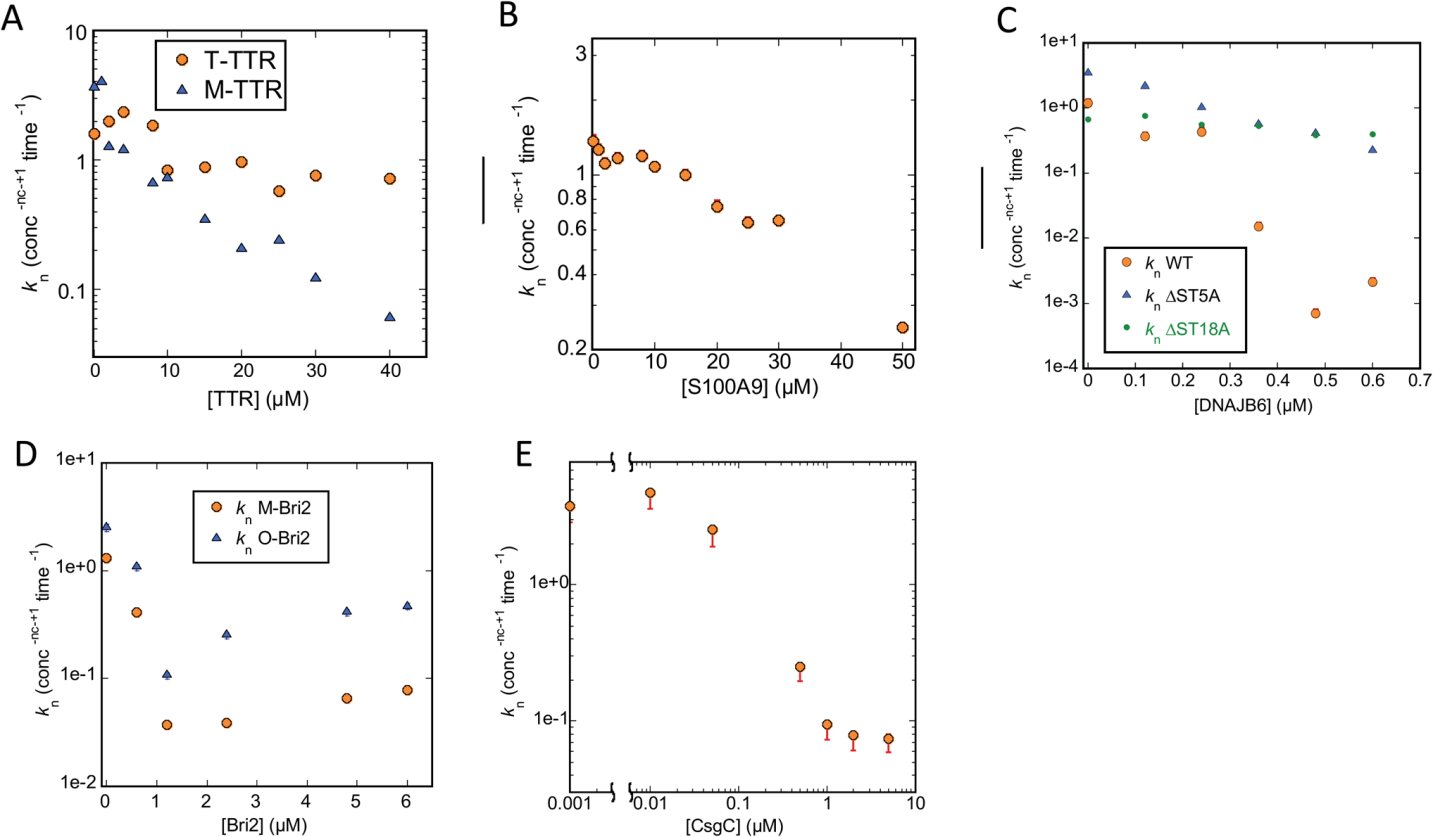
Primary nucleation rate constant *k*_n_ of FapC aggregation plotted against chaperone concentration. Values for *k*_n_ obtained from the best fit obtained by varying only *k*_n_ for the FapC aggregation curves in the presence of different chaperones (raw ThT curves presented in Fig. S4–S6†). Chaperones involved were (A) T-TTR and M-TTR, (B) S100A9, (C) DNAJB6 WT and the truncation mutants ΔST5A and ΔST18A, (D) monomeric-Bri2 and oligomeric-Bri2 and (E) CsgC.

Turning to Amylofit analysis of unseeded FapC fibrillation, the overall observation is once again that variation of *k*_n_ provides the best fit to the kinetic data (results in Fig. 8, fitted time curves in Fig. S4–S6†). This is also the case with T-TTR (Fig. S4A, D and G†) and M-TTR (Fig. S4B, E and H†) which are more efficient at inhibiting FapC than CsgA. Particularly for M-TTR, there is a marked difference in quality of fits and a very convincing log-linear relationship between *k*_n_ and [M-TTR] with a ∼10-fold decline in *k*_n_ as [M-TTR] increases to 40 μM (Fig. 8A). With S100A9, the fit with *k*_n_ is superior to that with *k*_2_ at the lowest concentration, although variation in either *k*_n_ or *k*_2_ satisfactorily fits data and scale log-linearly with [S100A9] with the same slope (Fig. 7C and S4† CFI). However, also with DNAJB6, *k*_n_ variation is strikingly better than *k*_2_ and *k*_+_ at fitting to the data, and *k*_n_ decreases much more with WT-DNAJB6 than the attenuated mutants (Fig. 7B and S5A–S5I†). M-Bri2 and O-Bri2 effects, though slightly irregular, are overall fitted much better by *k*_n_ variation (Fig. 7D, S6A, D, G and S6B, E, H†). CsgC is also fitted best by *k*_n_ variation though the variation is not linear in either a semi-log or log–log plot (Fig. 7E and S6C, F, I†).

**Fig. 9:**
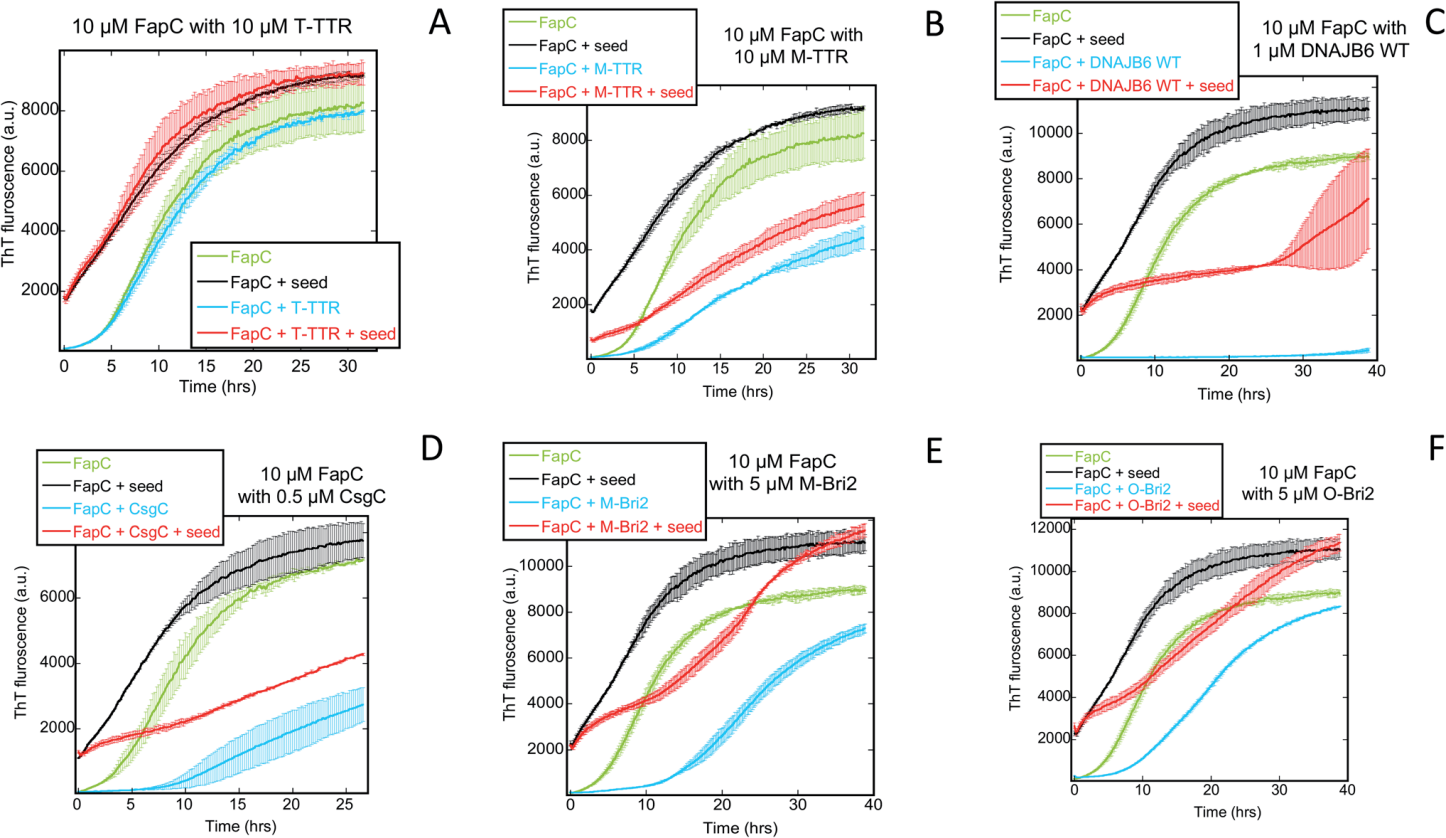
Seeding experiments with 10 μM FapC monomers using 2 μM FapC seeds (in monomer units) in the presence or absence of the indicated concentrations of different chaperones. At these chaperone concentrations, there is significant inhibition of aggregation, but not complete repression (Fig. 2). The chaperones were (A) T-TTR, (B) M-TTR (C) DNAJB6 WT, (D) CsgC, (E) M-Bri2 and (F) O-Bri2. Controls include FapC alone, FapC in the presence of chaperone without fibrils and FapC in presence of seeds/fibrils without chaperones.

FapC seeds eliminate the lag time of fibrillation. When the seeding experiment is repeated in the presence of chaperones, T-TTR (Fig. 9A) and S100A9 (Fig. S7†) have no effect, whereas the remaining 5 chaperones lead to significantly slower growth (Fig. 9B–F). This suggests that these chaperones also affect elongation and secondary nucleation although to a smaller degree. While these phenomena can occur in the absence of seeding, their effect becomes easier to detect when the primary nucleation step is eliminated by providing seeds.

The results of all these analyses are summarized in Table 1, which also shows that the chaperones inhibit spontaneous fibrillation of CsgA more than that of FapC, although the seeded fibrillation of FapC is inhibited more than that of CsgA.

**Table 1:** Summary of the impact of chaperones on the spontaneous and seeded fibrillation of CsgA and FapC

Chaperone	CsgA		FapC	
	Most affected aggregation step (fold reduction)	Effect of seeding	Rate constant most affected (fold reduction)	Effect of seeding
TTR	No effect	No effect	No effect	No effect
M-TTR	Modest effect; probably primary nucleation (×6 at 2 μM)	No effect	Primary nucleation (×10 at 40 μM)	Slower growth
S100A9	Modest effect; probably primary nucleation (×20 at 4 μM)	—	Primary (×6 at 50 μM) or secondary nucleation	No effect
Bri2 monomer	Primary nucleation (∼×10^3^ by 1 μM)	—	Primary nucleation (∼×40 by 6 μM)	Slower growth
Bri2 oligomer	Primary nucleation (∼×10^5^ by 1 μM)	—	Primary nucleation (∼×10 by 6 μM)	Slower growth
DNAJB6	Primary nucleation (∼×10^7^ by 0.12 μM)	No effect	Primary nucleation (∼×10^3^ by 0.6 μM)	Slower growth
CsgC	Primary nucleation (∼×10^5^ by 1 μM) and elongation	Very little effect	Primary nucleation and elongation	Slower growth

### The chaperones recognize monomeric FuBA but with varying affinities

**Fig. 10:**
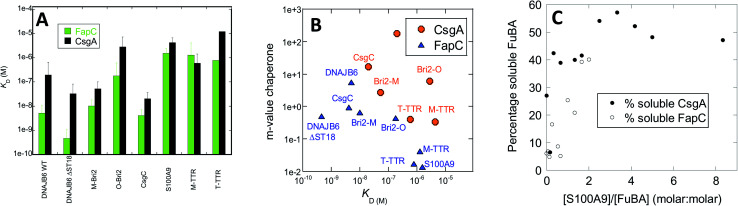
(A) Affinity constants of chaperones for monomeric CsgA and FapC obtained from Biacore experiments (raw data in Fig. S3†). (B) *m*-values of inhibition (obtained from slopes of log *k*_n_*versus* [chaperone], Fig. 3) plotted *versus* affinity constants originally shown in panel A. (C) Percentage non-aggregated CsgA and FapC as a function of molar ratio of S100A9. Based on SDS-PAGE gels in Fig. S4.†

Chaperone effects must involve interactions with the aggregating proteins, and the preferential targeting of primary nucleation implies binding to either monomers or small aggregates at the early stages of aggregation. To probe the affinity of interactions between FuBA and different chaperones, we immobilized FapC and CsgA on individual chips and measured the kinetics of binding and dissociation of chaperones at various concentrations (raw data in Fig. S8A–S8O,† results in Fig. 10A).

Both immobilized proteins were freshly desalted prior to immobilization, implying that they are largely monomeric though small aggregates may still have had time to form. Note that the initial parts of the curve are not fitted, as they represent bulk transport in connection with a change in buffer. Fitting of individual runs to obtain observed rates of association *k*_obs_ was followed by linear fits to extract rate constants of association and dissociation and thus dissociation constants *K*_D_ (examples provided in Fig. S8N and O†). This led to *K*_D_ values ranging over 3 orders of magnitude for both CsgA and FapC, as summarized in Table 1. Broadly speaking, FapC and CsgA showed the same variation in affinity towards the chaperones, with the lowest *K*_D_ values towards DNAJB6, Bri2 and CsgC and high values towards S100A9, M-TTR and TTR. This agrees well with the general observation that S100A9, M-TTR and TTR need to be present at concentrations up to 50 μM to have a significant impact on fibrillation of the bacterial amyloids, whereas DNAJB6, Bri2 and CsgC are effective at concentrations at or below 1 μM. More quantitatively, linear plots of log *k*_n_*versus* chaperone concentrations (as shown in Fig. 6 and 8) yield slopes whose magnitude (fold reduction in log *k*_n_ per unit chaperone, here termed *m*-value) can be seen as a measure of the chaperone efficiency of inhibition. The slope values are generally higher for CsgA, *i.e.* the chaperones inhibit CsgA more efficiently than FapC. For FapC there is a reasonable correlation between these slopes and the *K*_D_ values obtained from Biacore analysis (Fig. 10B), the outlier being DNAJB6-ΔST18A. The relationship is less significant for CsgA, although there is a trend that lower slope values (*i.e.* lower inhibition efficiency) accompany lower affinity (higher *K*_D_) for monomers. This is consistent with the model that these chaperones target monomeric species of FapC or CsgA (or early aggregates which have monomer-like structure) to prevent nucleation and thus fibrillation.

Interaction with monomeric FuBA and inhibition of aggregation implies that the chaperones keep FuBA soluble. To query this, we incubated CsgA and FapC with 0–50 μM S100A9 for 33 h at 37 °C, and analysed the amount of soluble CsgA/FapC by SDS-PAGE (Fig. S9†) (aggregated CsgA does not dissociate in SDS-PAGE loading buffer unless high concentrations of formic acid are present^57^). Using ImageJ to quantify band intensity and normalize relative to the original amount of soluble material, we note that S100A9 maintains a high solubility of 6 μM CsgA at sub-stoichiometric ratios (Fig. 10C). We see a weaker but still significant effect on FapC, whose solubility does not increase as steeply with increasing ratios of S100A9 (Fig. 10C). It is remarkable that S100A9 is able to maintain both CsgA and FapC in the monomeric state after such a long period of incubation. Potent aggregation-inhibitors such as EGCG and other polyphenols, while inhibiting amyloid formation for both CsgA^58^ and FapC,^59^ still lead to formation of higher-molecular weight SDS-resistant complexes^58^ which over 24 h form insoluble species that cannot be mobilized to migrate on gel filtration columns.^59^ Thus chaperones have an effect on CsgA and FapC which go mechanistically well beyond the effects of small-molecule inhibitors.

### Peptide array analysis reveals chaperone binding hot spots in both CsgA and FapC

We attempted to detect soluble complexes between functional amyloid and chaperones but were unsuccessful due to low concentrations of available material (data not shown). Instead, we used a peptide array displaying 14-mer peptides corresponding to different parts of the CsgA and FapC sequence to probe which parts of the FuBA sequence had higher affinity for the chaperones. The approach was aided by the fact that FuBA like CsgA and FapC are largely unfolded prior to aggregation, making peptide fragments reasonable representations of different parts of the monomeric state. Binding was quantified using fluorescently-labeled chaperones.

**Fig. 11:**
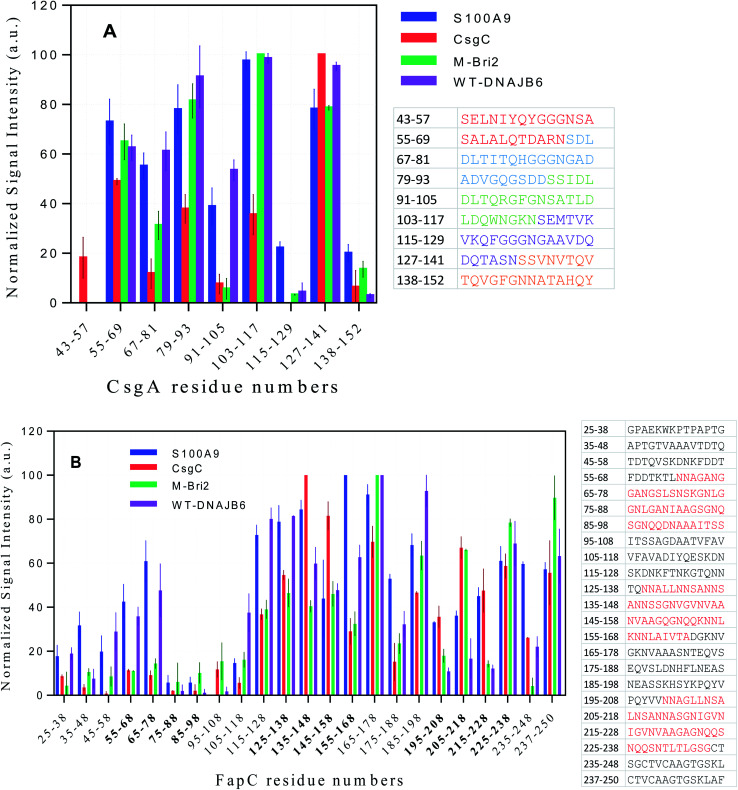
Interactions of four different fluorescent-labeled chaperones with peptide array spots displaying (A) CsgA and (B) FapC sequences. Chaperones are indicated in the graph legend. For each spot, the numbers on the *x*-axis state the residue position in the intact FapC or CsgA sequence corresponding to the start and end of residue in the spot's 14-mer peptide. For CsgA, the 5 imperfect repeats are indicated by different colors in the associated table, while FapC's three repeat sequences have a red color in the table and bold style on the *x*-axis.

Generally, the different chaperones tested show remarkably similar preferences for the different sequences both for CsgA and FapC. However, multiple regression analysis failed to find a correlation between signal intensity and peptide hydrophobicity^60^ or charge, either alone or in combination (data not shown). Thus, simple physical–chemical properties cannot explain the variation in binding preferences. Closer inspection revealed a tendency towards a binary pattern amongst the CsgA peptides (Fig. 11A), *i.e.* the 1^st^, 3^rd^, 5^th^, 7^th^ and 9^th^ peptides (those with sequences from one repeat only) are recognized poorly while the 2^nd^, 4^th^, 6^th^ and 8^th^ (straddling two repeats) are recognized better. This suggests that chaperones may recognize the existence of multiple co-existing β-turns. Alternatively, the variation could reflect more general aggregation tendencies.

To address this, we turned to the Rosetta program which predicts aggregation energies of hexapeptides (Fig. S10A†).^61^ A value of −23 kcal mol^−1^ is considered a threshold; lower values predict significant aggregation. The region containing residues 127–141 consists of hexapeptides with high aggregation propensity. It is noteworthy that there are three clusters of residues where at least 3 peptides are in close proximity to each other and exceed this threshold, namely 53–58, 108–112 and 128–136. All three clusters are found among the well-recognized peptides in Fig. 11A, suggesting that the chaperones recognize intrinsic aggregation propensities.

FapC peptides also show a significant variation in chaperone affinities across the sequence and markedly higher interaction around the R2 (residues 127–163), L2 (164–199), R3 (200–236) and the C-terminal region of FapC (Fig. 11B). The correspondence between these binding spots and the distribution of aggregation-prone peptide sequences according to Rosetta (Fig. S10B†) is less clear-cut than for CsgA. It remains unclear whether this reflects the greater complexity of FapC sequences, where repeat regions are separated by less well-conserved linker regions of variable sequence. However, we note that both Rosetta analysis and peptide displays focus on local sequences and should therefore not be sensitive to long-range effects.

### The most efficient chaperones reduce biofilm formation

**Fig. 12:**
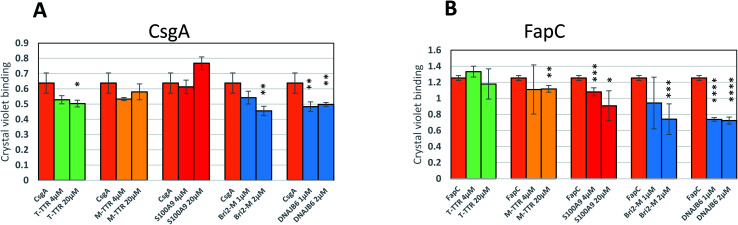
(A) and (B) Effect of different concentrations of chaperones on biofilm growth by *E. coli* and *Pseudomonas* expressing CsgA or FapC, respectively. Asterisks are used as follows: *: *p* < 0.05; **: *p* < 0.025; ***: *p* < 0.01; ****: *p* < 0.001.

Finally, to analyze the biological consequences of the chaperones' inhibitory activity, we let *E. coli* and *Pseudomonas* bacterial strains expressing CsgA and FapC respectively form bacterial biofilm in the absence or presence of different amounts of chaperones, using chaperone concentrations shown to be effective in the preceding *in vitro* assays. For CsgA, there is no significant effect by the weakly inhibiting chaperone S100A9. It has previously been reported that T-TTR only weakly inhibits biofilm formation, while M-TTR is much more potent, consistent with their capacities to inhibit fibrillogenesis. Bri2 and DNAJB6, the two most effective aggregation inhibitors (Fig. 12A) led to a significant reduction in biofilm. Similar results are obtained for FapC, where the effects of Bri2 and DNAJB6 are even more marked, leading to a *ca.* 40% reduction in biofilm formed (Fig. 12B). Thus, we conclude that inhibition of fibrillation has direct and deleterious consequences for biofilm formation.

## Discussion

### Chaperones work by targeting a common aggregation step though the targeted species may vary between monomer and nucleus

Chaperones can interact with a variety of aggregated species besides the monomeric state and thus regulate the concentration of aggregates at different stages of the process.^41,62^ Analysis of the kinetic profiles of aggregation of functional amyloids CsgA and FapC in the presence of this set of chaperones using the methodology in Amylofit allows us to identify the microscopic processes and species most likely affected by the chaperone and quantify the extent of their impact on the associated rate constants (Fig. 6 and 8). Chaperones present at concentrations high enough to suppress formation of ThT-positive aggregates generally lead to shorter, thinner and more curvy aggregates. However, our kinetic analysis shows that, as a group, the chaperones do not fundamentally change the mechanistic framework of aggregation of CsgA and FapC, *e.g.* by diverting aggregation to pathways dominated by secondary processes such as fragmentation or secondary fibrillation or other types of aggregating species. Rather, the chaperones primarily target one specific step, namely primary nucleation.

At face value, the simplemindedness of this approach contrasts with the chaperones' more diverse effects towards pathological proteins such as Aβ and the yeast prion protein Ure2p.^41^ DNAJB6 primarily inhibits the primary nucleation of Aβ, while both Bri2 and the Brichos chaperone (a close homologue to Bri2) targets secondary nucleation;^41^ in addition, Bri2 affects the elongation step. S100A9 increases the primary nucleation rate of Aβ42, but significantly decreases the rate of secondary nucleation due to templating of S100A9 fibrils on the surfaces of Aβ42 fibrils and coating the Aβ422 fibrillar surfaces with S100A9 amyloids.^48^

Nevertheless, our conclusion should be nuanced in several ways. Firstly, seeding experiments (Fig. 7 and 9) reveal that the chaperones are also able to impact other steps of the aggregation process and these effects, although perhaps modest by themselves, become more easy to detect if the presence of seeds bypasses the need for primary nucleation. This is particularly manifest with FapC, where the 3 strongest chaperones (DNAJB6, CsgC and Bri-2) all reduce the fibrillation growth rate. This indicates that they must also have a certain affinity for later aggregation species, which is only eclipsed by their ability to target the nucleus or the monomer. Secondly, the identification of the primary nucleation step as the target of intervention does not in itself establish the main binding target for the chaperone; this could be either the monomer or the oligomer. Here stoichiometric information can provide insight. Only CsgC and DNAJB6 are effective at substoichiometric molar ratios (Fig. 1F–I and 2F–I). This implies that these chaperones target species present at concentrations well below that of the monomer, *i.e.* the nucleus itself. By a similar rationale, DNAJB6 is proposed to target the oligomeric or nucleated state of Aβ rather than the monomeric species^46^ and thus appears to maintain this strategy irrespective of client. Remarkably, both CsgC and DNAJB6 are *bona fide* chaperones dedicated to the rescue of specific aggregation-prone proteins. The other chaperones are not streamlined in the same manner. Neuronal T-TTR expression has been shown to be regulated by HSF1, thus its interaction with Aβ is likely to reflect a programmed chaperone response to neuronal stress in which it can respond to exposed hydrophobic regions of misfolded proteins.^63^ The physiological or proteostatic role of M-TTR *in vivo* is unclear. S100A9 has other major physiological roles which allow it to connect amyloid self-assembly with inflammation (the amyloid-neuroinflammatory cascade^64–66^) and its chaperone activity may be an instance of molecular moonlighting. Bri2 very clearly prefers aggregates of Aβ, but may likely target monomers of CsgA and FapC in the aggregation process (unless presented with preformed aggregates). The stoichiometry of Bri-2 effects (where effects become significant around 1 : 1 molar ratios, Fig. 1D, E and 2D, E) makes it unclear whether monomers or nuclei are the preferred species, however.

### Structural basis for the targeting

What could be the reason for the apparent preference for inhibiting primary nucleation of functional amyloids? Both CsgA and FapC are both known to favour a fast track in aggregation, dominated by primary nucleation and elongation^51^ and relegating secondary processes to a minor track, unless provoked by the removal of one or more of the imperfect repeats.^52^ Thus targeting primary nucleation is the most efficient way to inhibit aggregation. FapC is generally less sensitive to chaperones than CsgA. There are several possible reasons for this. Firstly, FapC is a larger protein than CsgA, while the chaperones tested here have similar molecular sizes to CsgA; this may help them to more effectively block the initial nucleus from further growth. Secondly, FapC possesses a long linker/loop region between imperfect repeats, which could repel or block effective binding of the chaperone. In both cases, however, the protein is recognized quite efficiently in the monomeric state by chaperones and the efficiency of inhibition scales roughly with the affinity of binding; furthermore, there are certain hotspots within the protein sequence, which may relate to the aggregation propensities and in this way reflect the modular build-up of the functional amyloid proteins. Thus, the very uniformity and simplicity of functional amyloid aggregation makes it a strategic target for chaperones and in this way more susceptible to biological control, in line with its recruitment for useful biological properties. In contrast, it could be speculated that chaperones show varying mechanisms against different pathogenic amyloid precursor proteins because they aggregate by a greater diversity of mechanisms than do the functional amyloids.

### Biological and therapeutic perspectives

What makes for the best aggregation inhibitor? The aggregation nucleus is the bottleneck, through which all amyloid-forming molecules must pass in the absence of seeds. Targeting this species is stoichiometrically the most efficient way to nip the burgeoning aggregate in the bud. However, once aggregates have started to elongate, targeting the nucleus becomes an increasingly irrelevant strategy to suppress aggregation. Since fibrils can mature by other approaches such as elongation, secondary nucleation and fragmentation, a molecularly flexible approach, capable of recognizing diverse sites and modes of aggregation growth may be the best way to control unwanted self-assembly.

For proteins such as functional amyloid, which largely rely on primary nucleation and elongation, inhibition of nucleation will have greater impact than for proteins with multiple different aggregation mechanisms. The fact that efficient chaperones can reduce biofilm growth when added exogenously suggests that nucleus-like species are being formed by functional amyloid on the bacterial surface. DNAJB6 is able to inhibit biofilm growth despite its inability to stop elongation of existing fibrils *in vitro*. Perhaps it acts in the early stages of bacterial amyloid growth at the bacterial cell surface by interfering with the binding of CsgA or FapC to nucleator proteins such as CsgB or FapB. Accordingly it may be useful simply to have the chaperone available at potential sites of aggregation. S100A9 is abundant at sites of inflammation which are also potential entry sites for bacteria, thus fibrillation can potentially be blocked from the onset by S100A9. Further insight into these aspects will be aided by the ability to detect amyloid formation in real time in biofilm using amyloid-specific fluorophores such as oligothiophenes.^67^

From the bacterial perspective, it is only relevant to prevent aggregation of functional amyloid prior to the actual export to the outside of the outer membrane. During passage across the periplasmic space, the protein must remain monomeric to allow passage through the channel in the outer membrane and presentation to either the nucleator protein or the growing ends of the fibrils. It makes little evolutionary sense to inhibit aggregation once functional amyloid formation has started outside the cell, since the greatest benefit comes from having long, extended fibrils. The lack of secondary nucleation also suggests that the sides of the fibrils are not productive sites for binding. This makes physical sense, since long fibrils will be more likely to entangle and stabilize the biofilm with ensuing increase in mechanical stability.^10^

While it has therapeutic potential to design molecules to target the initial nucleus, a broad range amyloid aggregation inhibitor would most likely have greater impact. As always in biology it is a question of developing strategies to recognize binding sites that retain high affinity for the targeted sites along with broad specificity for multiple species. Many chaperones' broad range of substrates but relatively weak affinity represents one way of dealing with this problem. This feature likely requires a versatile and extensive binding surface which is difficult to recapitulate in small molecules unless augmented by *e.g.* supramolecular assembly of multiple functional groups.

## Materials and methods

### Recombinant protein expression and purification

Chaperones were purified as described for TTR, M-TTR,^34^ S100A9,^37^ Bri 2,^43^ DNAJB6 (ref. 46 and 68) and CsgC.^69^ The DNAJB6 mutant F91L was used as pseudowildtype as it shows wildtype properties in its general chaperone activities (C.E., unpublished observations). Full-length FapC (residues 25–250) from *Pseudomonas* sp. UK4 and CsgA from *E. coli* (residues 21–151), both without their signal sequences, were expressed from pET28d and pET11d vectors with Kanamycin and Ampicillin as antibiotic markers as described.^52,70^ Briefly, bacterial plasmids were transformed into BL21 *E. coli* bacteria and plated on LB-agar. A single colony from freshly transformed plates was inoculated into 50 ml liquid medium, grown overnight, and used as a 40-fold diluted inoculum, leading to a starting OD_600_ of 0.1 in the main culture. Protein expression was induced at OD_600_ 0.6–0.8 with 0.5 or 1 mM IPTG for CsgA and FapC, respectively. Cells were harvested 12 or 4 h later for CsgA and FapC, respectively and spun down (6000*g* for 10 min at 4 °C). The cells were lysed in buffer A (50 mM Tris pH 7.5 and 8 M guanidinium chloride (GuCl)). The cleared lysate was loaded on a His-Tag affinity column. To maximize elution, target protein was eluted in two step gradients (300 mM and 500 mM imidazole in buffer A). The purified proteins were used immediately (prolonged GuCl incubation leads to atmospheric oxidation, which reduces aggregation^71^). Proteins were desalted into 50 mM Tris pH 7.5 on a PD-10 column (GE Healthcare) containing Sephadex G-25 beads according to the manufacturers' protocols.^70,72^ All subsequent reactions were carried out in 50 mM Tris pH 7.5 unless otherwise specified.

### Preparation of amyloid seeds/fibrils for seeding assays

Freshly desalted protein at 0.2–0.4 mg ml^−1^ was incubated in a rotating shaker (35 rpm) for at least 48 h at RT (25 °C). To remove soluble material, fibrils were subjected to 3 cycles of centrifugation (20 800*g*, 5 min, RT) and resuspension of the pellet. Finally, the fibrils were sonicated in a water bath for 15 min. Sonicated fibrils were used immediately after preparation.

### Thioflavin T (ThT) fluorescence assay

Aggregation kinetics were monitored in 96 well plates (Corning, Flat Bottom, Non-Binding Surface, Non-Sterile, Black Polystyrene) through the increase in fluorescence accompanying binding of the dye ThT to fibrils. Each well contained 200 μl of sample, consisting of 10 μM ThT, 1–10 μM freshly desalted CsgA or FapC and variable amounts of chaperones. Seeding experiments were carried out using 1 μM CsgA or 10 μM FapC supplemented with either 50 nM CsgA seeds (in monomer units) or 2 μM FapC seeds (monomer units). For these seeding experiments, chaperone concentrations were chosen that led to retardation but not suppression of aggregation (see Fig. 7 and 9 for details). ThT fluorescence intensity was monitored in a plate reader (Clariostar, BMG Labtech, Germany) every 10 min at 25 °C at 485 nm after excitation at 448 nm. Multiple readings were obtained per well (spiral scan with scan diameter of 5; values were averaged to get the final value). The plate was shaken (double orbital) for 2 s before every measurement. The gain was set to 1500 with a focal height of 4.

### Transmission electron microscopy (TEM) and negative staining

Fibrillated samples from the end point of the ThT aggregation assay (∼5 μl) were applied onto a glow-discharged 400-mesh carbon-coated copper grids for 60 s. The grids were then flash washed by blotting to remove excess fibrils and stained with a drop of 2% (w/v) aqueous uranyl acetate for 60 s. Excess stain was removed by blotting with filter paper. The fibril-containing grids were air-dried and imaged on a Tecnai™ G2 Spirit transmission electron microscope, operated at 80 kV. Digital acquisitions were performed with a bottom mounted Tietz camera cooled to 0 °C.

### AFM imaging

20 μl of each sample, supplemented with 2 μl 10 mM HCl were deposited on a mica surface for 30 minutes and washed 5 times with 200 μl deionized water before being dried overnight at room temperature. AFM imaging was performed on a BioScope Catalyst atomic force microscope (Bruker), in peak force tapping mode in air. Resolution was set at 512 × 512 pixels, scan rate was 0.51 Hz, and scan sizes were 2 × 2 and 5 × 5 μm. Bruker SNL10 cantilevers were used for all measurements.

### SDS-polyacrylamide gel electrophoresis

Reducing SDS-PAGE^73^ was used to determine the amount of non-amyloid FuBA protein present at the end point of the ThT aggregation assay. 10 μl of protein sample was mixed with 10 μl SDS-sample buffer, boiled for 5–10 min at 95 °C and electrophoresed on gels consisting of a 5% (w/v) stacking gel and 12% (w/v) separating gel at 200 volts per gel for 60 min. Commercial Mini-Protean™ BIORAD 4–12% Bis-Tris Midi Gels (BIORAD, 1.0 mm × 15 well) were run as instructed by the manufacturers. Unless otherwise stated, 10 μl were loaded per lane. Gels were stained with Coomassie R-250. Band intensities were quantified using Image J.

### SPR analysis

SPR assays were carried out on a Biacore 2000 system (GE Healthcare). Freshly desalted CsgA and FapC were immobilized on a CM5 sensor chip using amine coupling chemistry. Running buffer contained 10 mM HEPES pH 7.4, 150 mM NaCl, 3 mM EDTA and 0.05% BSA. Chaperones were injected at different serial dilutions (DNAJB6, Bri and CsgC: 10–2000 nM; S100A9, TTR and MTTR: 5–70 μM). All chaperones were passed over immobilized CsgA, FapC and a blank surface control at 20 μl min^−1^. Data were fitted with a 1 : 1 binding model using Biacore Evaluation Software (GE Healthcare).

### Biofilm studies

A single bacterial colony was transferred to LB medium with appropriate antibiotics (Table 2) and grown overnight at 28 °C, 180 rpm. The culture was then diluted to OD_600_ ∼0.5 with fresh liquid media and 160 μl culture was transferred to each of the 96 wells. Peg lids (Nunc™ 445497 Immuno™ TSP Lids) were inserted into the 96 plate wells for 1 hour to initiate attachment and biofilm growth on the peg surface. The lids were then transferred to another 96 well plate with 160 μl of fresh LB medium per well and either 0, 4 or 20 μM of various chaperones and incubated for 48 h to allow growth of biofilm on the pegs. To quantify biofilm, peg lids were washed in MilliQ to remove planktonic bacteria, dried for 1 h at room temperature and then submerged in a 96 well new plate with 160 μl per well of Gram's crystal violet solution for 15 min. To remove excess stain, the peg lid was washed twice with mQ water. Finally, bound crystal violet was released from the biofilm by incubation of the peg lids in 33% v/v acetic acid (glacial acetic acid diluted with MilliQ water) for 30 min at room temperature and absorption at 590 nm measured. At least 3 biological replicates were included. Bacteria-free wells were used to measure the background level of crystal violet. This value was subtracted from biofilm absorption values.

**Table 2:** List of strains used for biofilm studies

Strain	Phenotype	Antibiotics
CsgA SM2258 (ref. 74)	Curli overexpressing	Tetracycline
CsgA SM2257 (ref. 74)	Curli negative	Kanamycin
FapC wt+^59^	FapC *Pseudomonas* sp. UK4 overexpressing	Ampicillin
FapC Δfap^59^	FapC *Pseudomonas* sp. UK4 negative	Gentamycin

### Peptide arrays

Peptide microarrays containing FapC and CsgA sequences were used to probe the interaction of each chaperone with FapC/CsgA. The array contained immobilized 14-residue peptides^75^ from the FapC/CsgA sequence, each peptide spot displaced forward by 10 residues compared to the preceding peptide (*i.e.* 4 residues of overlap). Chaperones S100A9, CsgC, DnaJ (wt) and DnaJ-F91L were labeled with Alexa Fluor 546 (A546; Thermo-Fisher, Waltham, MA) and Bri2 monomer was labelled with Fluidiphore 503 dye according to the manufacturer's protocols (Fluidic Analytics, Cambridge UK). Free A546 was separated using PD-10 desalting column (GE Life Sciences), while free Fluidiphore 503 was removed by centrifugation (13 000 rpm, 10 min). The microarray was first blocked with TRIS saline buffer containing 0.1% Tween-20 (TSB-T) and 3% (w/v) whey protein at 4 °C overnight, washed three times with TSB-T and incubated with 0.05 mg ml^−1^ of labelled chaperones for 4 hours at room temperature. After three times washing of the microarray with TSB-T, the arrays were air-dried in the dark and scanned using a Typhoon Trio scanner (GE Life Sciences, Pittsburgh, PA). Spot intensities were quantified using ImageJ.

### Kinetic modelling analysis

The AMYLOFIT web interface was used to fit the aggregation curves to determine molecular mechanisms of the inhibition of amyloid aggregation.^50^ ThT aggregation kinetics acquired in the presence of increasing amounts of chaperone were analysed using a secondary nucleation model.^72^ Kinetic curves of chaperone inhibition were fitted to obtain rate constants for primary nucleation (*k*_n_), elongation (*k*_+_), and secondary nucleation (*k*_2_) at different chaperone concentrations. All fitting used a 3 basin hop algorithm with errors and normalized ThT kinetics data as described.^50^ To determine the most critical step affecting the aggregation, each parameter (*k*_n_, *k*_+_ and *k*_2_) was individually varied, while keeping the others as global constant. The best fit was the one with the lowest mean squared residual error (MRE) value.

## Data availability

All data are provided in figures and tables. Raw data can be provided upon request.

## Author contributions

M. N. and D. E. O: conceptualization, methodology, formal analysis, investigation, writing-original draft and editing. D. E. O.: project administration, funding acquisition and supervision. J. B.: resources, writing-original draft and editing. Z. N.: methodology and formal analysis. J. P: investigation. L. M.-R: resources and editing. H. B., G. M., V. S., S. M., C. E., J. J.: resources.

## Conflicts of interest

The authors declare that they have no competing interests.

## Supplementary Material

SC-013-D1SC05790A-s001
