# Supplementary material for: Chaperones mainly suppress primary nucleation during formation of functional amyloid required for bacterial biofilm formation
Source: Chem Sci. 2021 Dec 13;13(2):536–53. doi: 10.1039/d1sc05790a (PMC8729806; doi:10.1039/d1sc05790a)
Supplement: SC-013-D1SC05790A-s001 [file SC-013-D1SC05790A-s001.pdf]

Electronic Supplementary Material (ESI) for Chemical Science.
This journal is © The Royal Society of Chemistry 2021

Chaperones mainly suppress primary nucleation during formation of functional amyloid required for bacterial biofilm formation

Madhu Nagaraj, Zahra Najarzadeh, Jonathan Pansieri, Henrik Biverstål, Greta Musteikyte, Vytautas Smirnovas, Steve Matthews, Cecilia Emanuelsson, Janne Johansson, Joel N. Buxbaum, Ludmilla Morozova-Roche and Daniel Otzen

Supplementary Information

Nagaraj et al Figure S1: Fits to CsgA

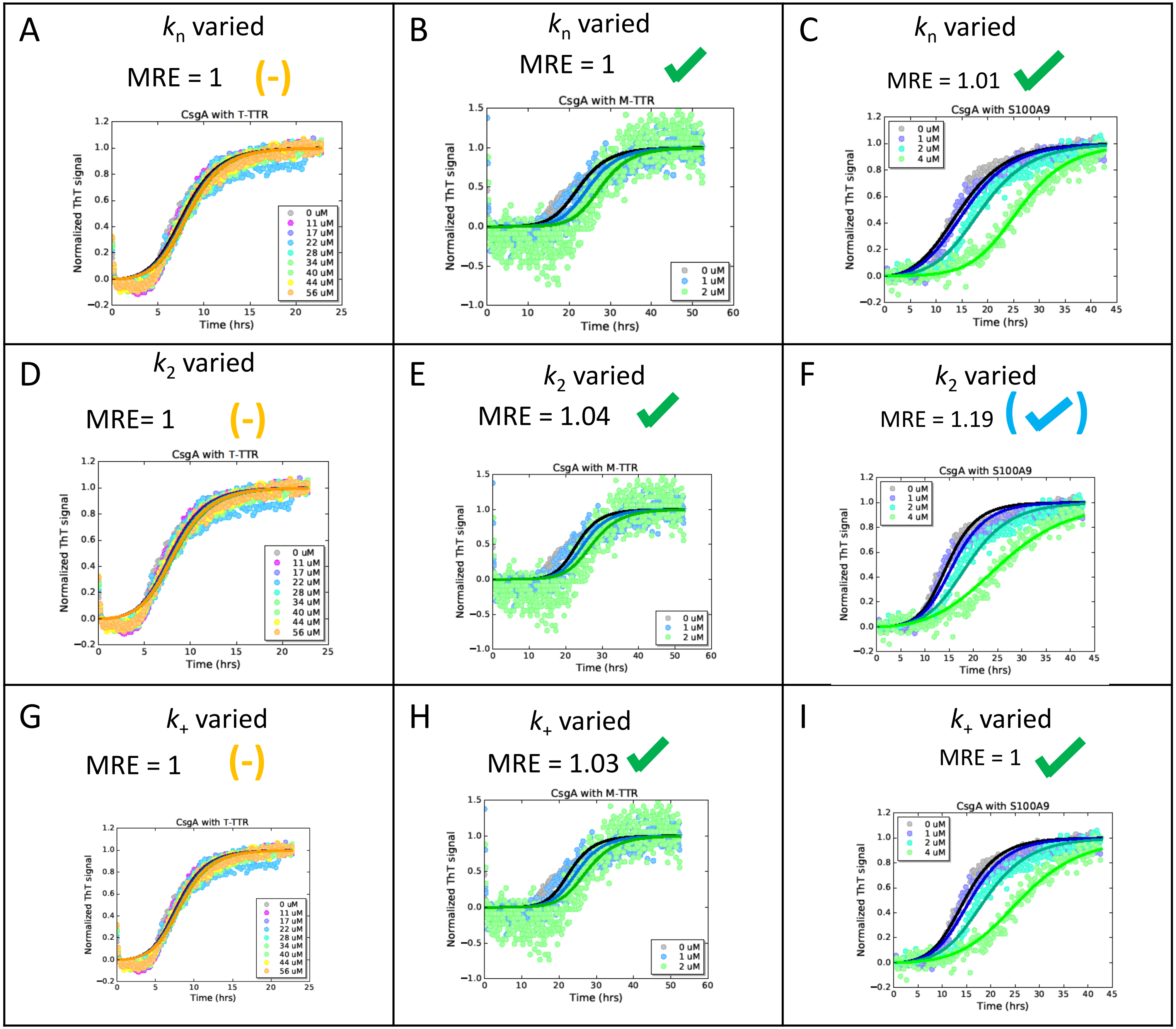

Figure S1. Fits to CsgA aggregation data in the presence of different concentrations of T-TTR, M-TTR and S100A9 (indicated in the graph panels), using the same model involving primary nucleation, elongation and secondary nucleation but allowing either k_n (ABC), k_2 (DEF) or k_+ (GHI) to vary. For each chaperone, the mean residual error is normalized to the fit with the lowest MRE value (shown as a tick mark: green tick shows the best fit, blue tick is intermediate, orange tick shows insufficient variation in time curves).

Nagaraj et al Figure S2: Fits to CsgA

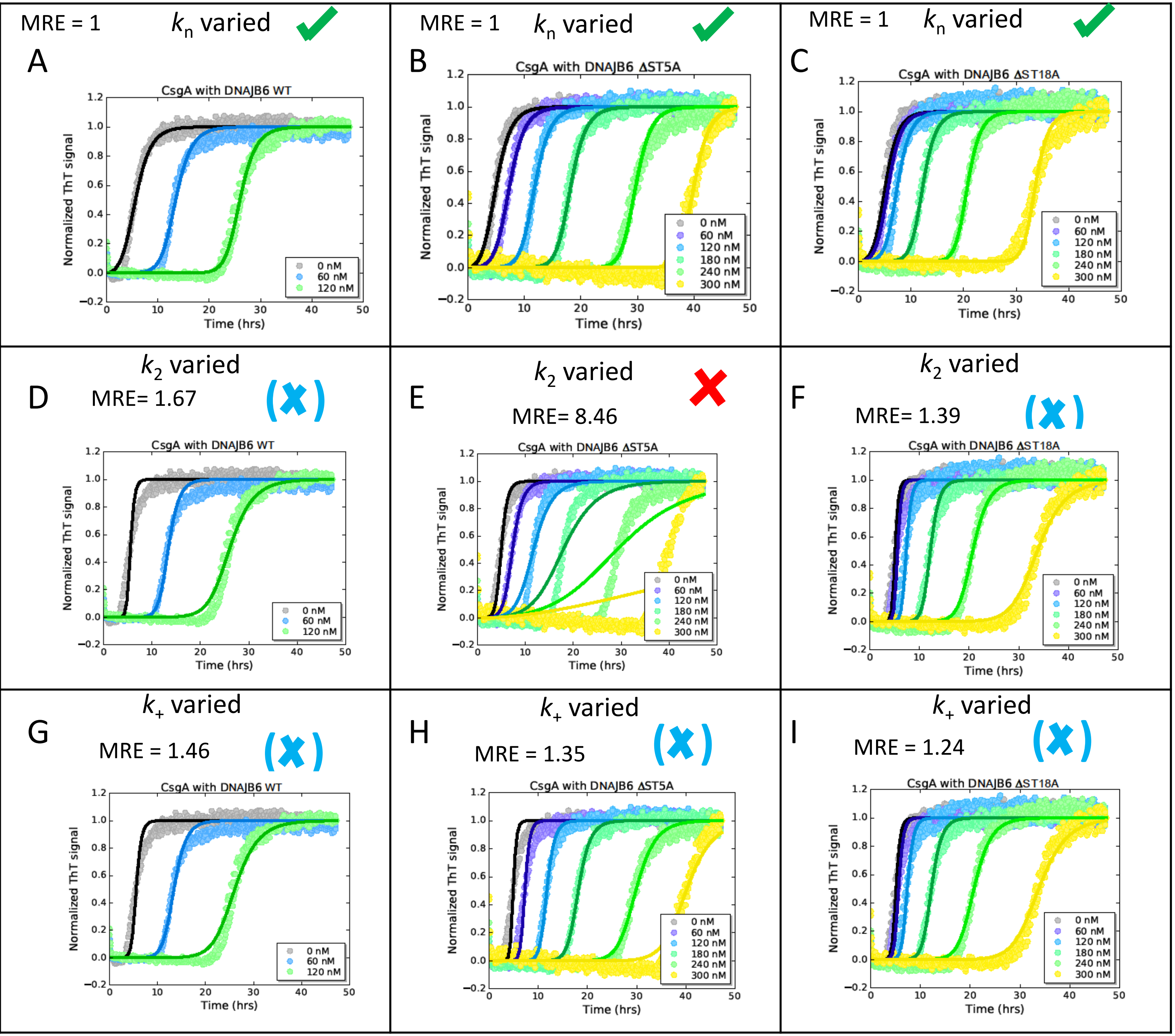

Figure S2. Fits to CsgA aggregation data in the presence of different concentrations of DNAJB6 WT, DNAJB6 ΔST5A, and DNAJB6 ΔST18A (indicated in the graph panels), using the same model involving primary nucleation, elongation and secondary nucleation but allowing either k_n (ABC), k_2 (DEF) or k_+ (GHI) to vary. For each chaperone, the mean residual error is normalized to the fit with the lowest MRE value (shown as a tick mark or cross).

Nagaraj et al Figure S3: Fits to CsgA

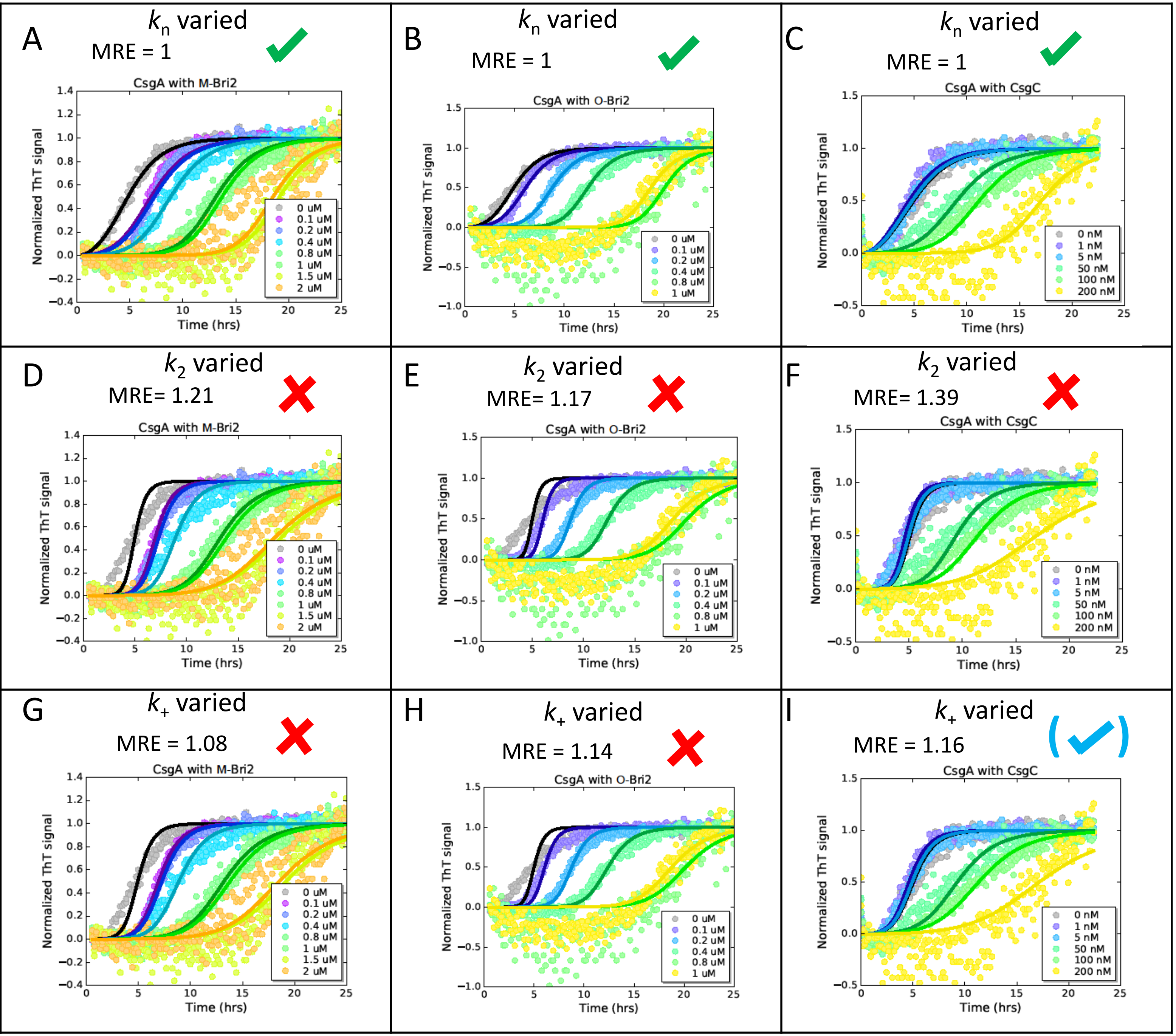

Figure S3. Fits to CsgA aggregation data in the presence of different concentrations of Monomeric-Bri2, Oligomeric-Bri2, and CsgC (indicated in the graph panels), using the same model involving primary nucleation, elongation and secondary nucleation but allowing either k_n (ABC), k_2 (DEF) or k_+ (GHI) to vary. For each chaperone, the mean residual error is normalized to the fit with the lowest MRE value (shown as a tick mark).

Nagaraj et al Figure S4: Fits to FapC

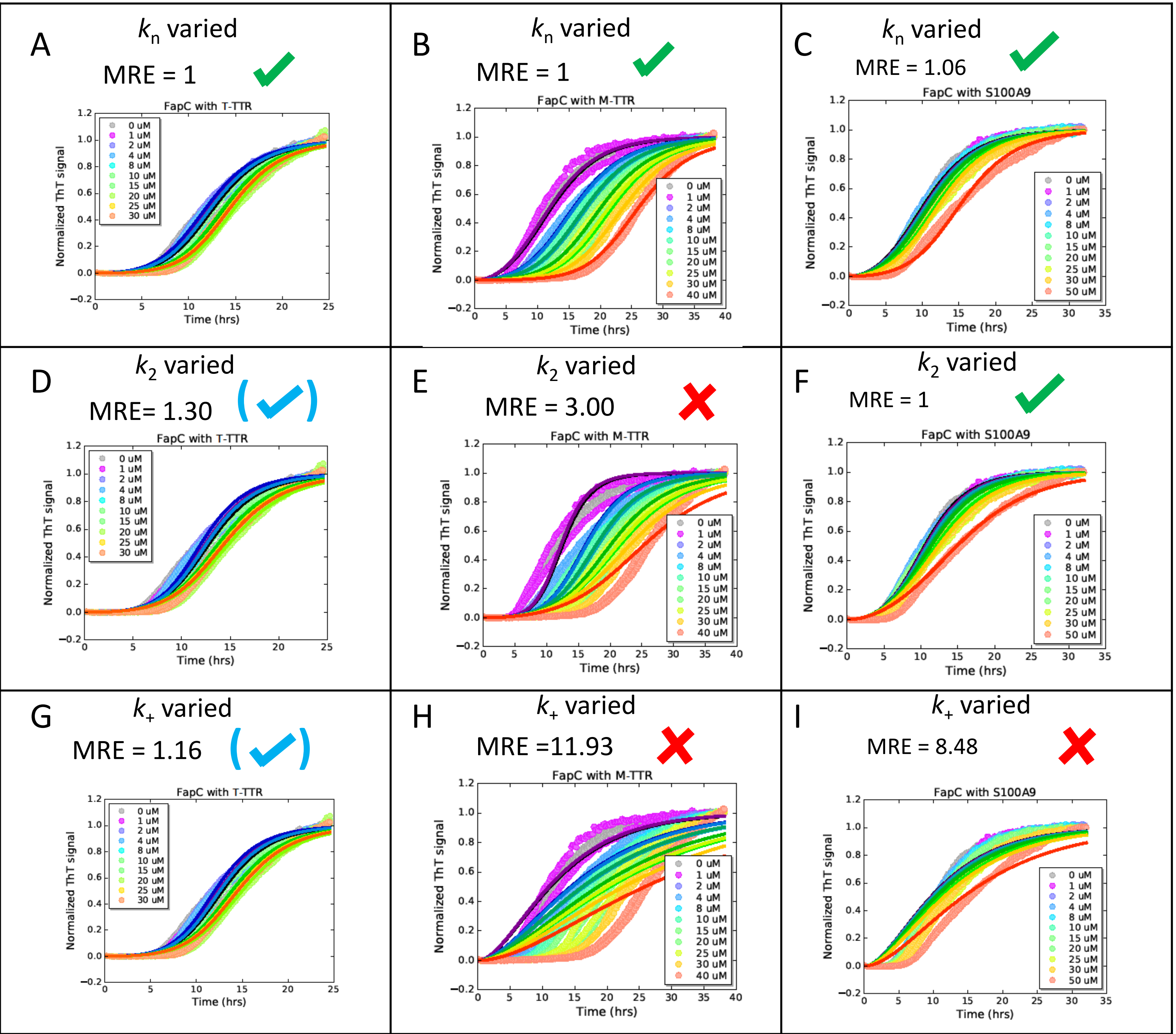

Figure S4. Fits to FapC aggregation data in the presence of different concentrations of T-TTR, M-TTR and S100A9 (indicated in the graph panels), using the same model involving primary nucleation, elongation and secondary nucleation but allowing either k_n (ABC), k_2 (DEF) or k_+ (GHI) to vary. For each chaperone, the mean residual error is normalized to the fit with the lowest MRE value (shown as a tick mark).

Nagaraj et al Figure S5: Fits to FapC

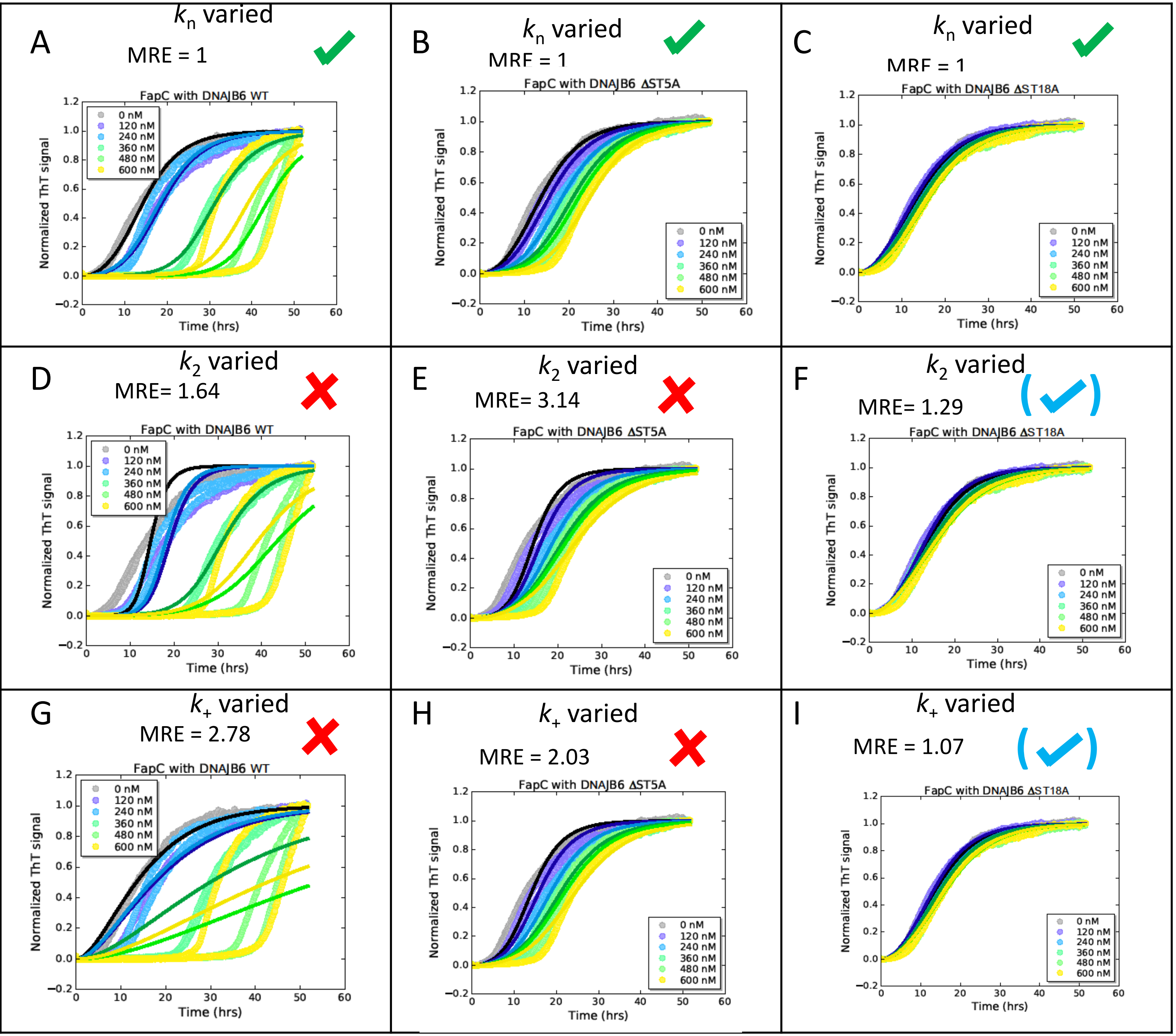

Figure S5. Fits to FapC aggregation data in the presence of different concentrations of DNAJB6 WT, DNAJB6 ΔST5A, and DNAJB6 ΔST18A (indicated in the graph panels), using the same model involving primary nucleation, elongation and secondary nucleation but allowing either k_n (ABC), k_2 (DEF) or k_+ (GHI) to vary. For each chaperone, the mean residual error is normalized to the fit with the lowest MRE value (shown as a tick mark).

Nagaraj et al Figure S6: Fits to FapC

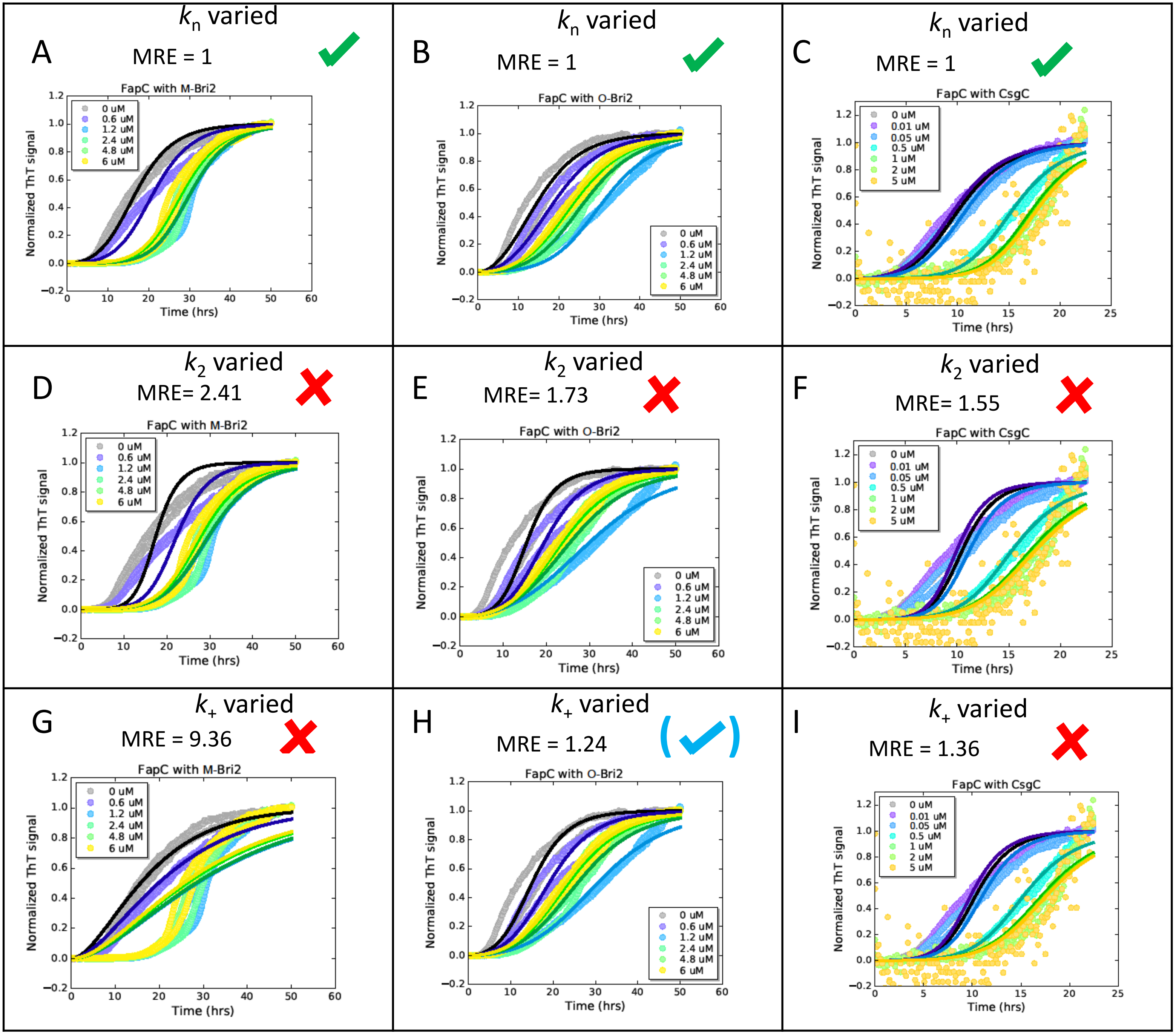

Figure S6. Fits to FapC aggregation data in the presence of different concentrations of Monomeric-Bri2, Oligomeric-Bri2, and CsgC (indicated in the graph panels), using the same model involving primary nucleation, elongation and secondary nucleation but allowing either k_n (ABC), k_2 (DEF) or k_+ (GHI) to vary. For each chaperone, the mean residual error is normalized to the fit with the lowest MRE value (shown as a tick mark).

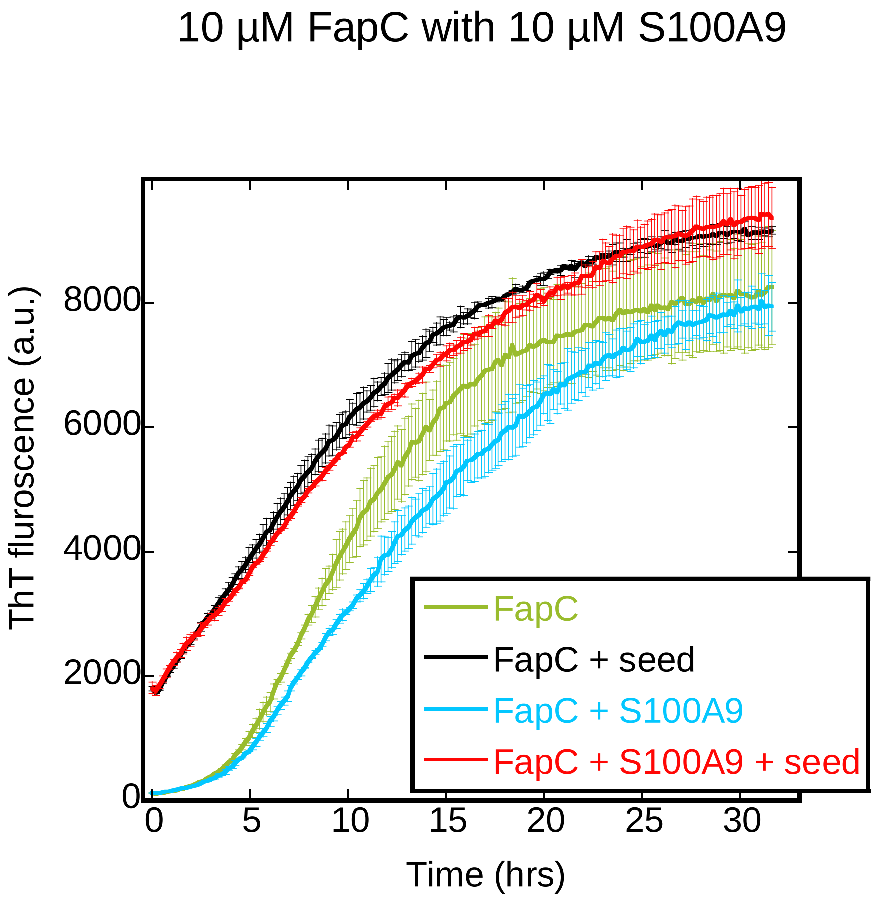

Figure S7. Seeding experiments with 10 µM FapC monomers and 2 µM FapC seeds (in monomer units) in the presence or absence of 10 µM S100A9.

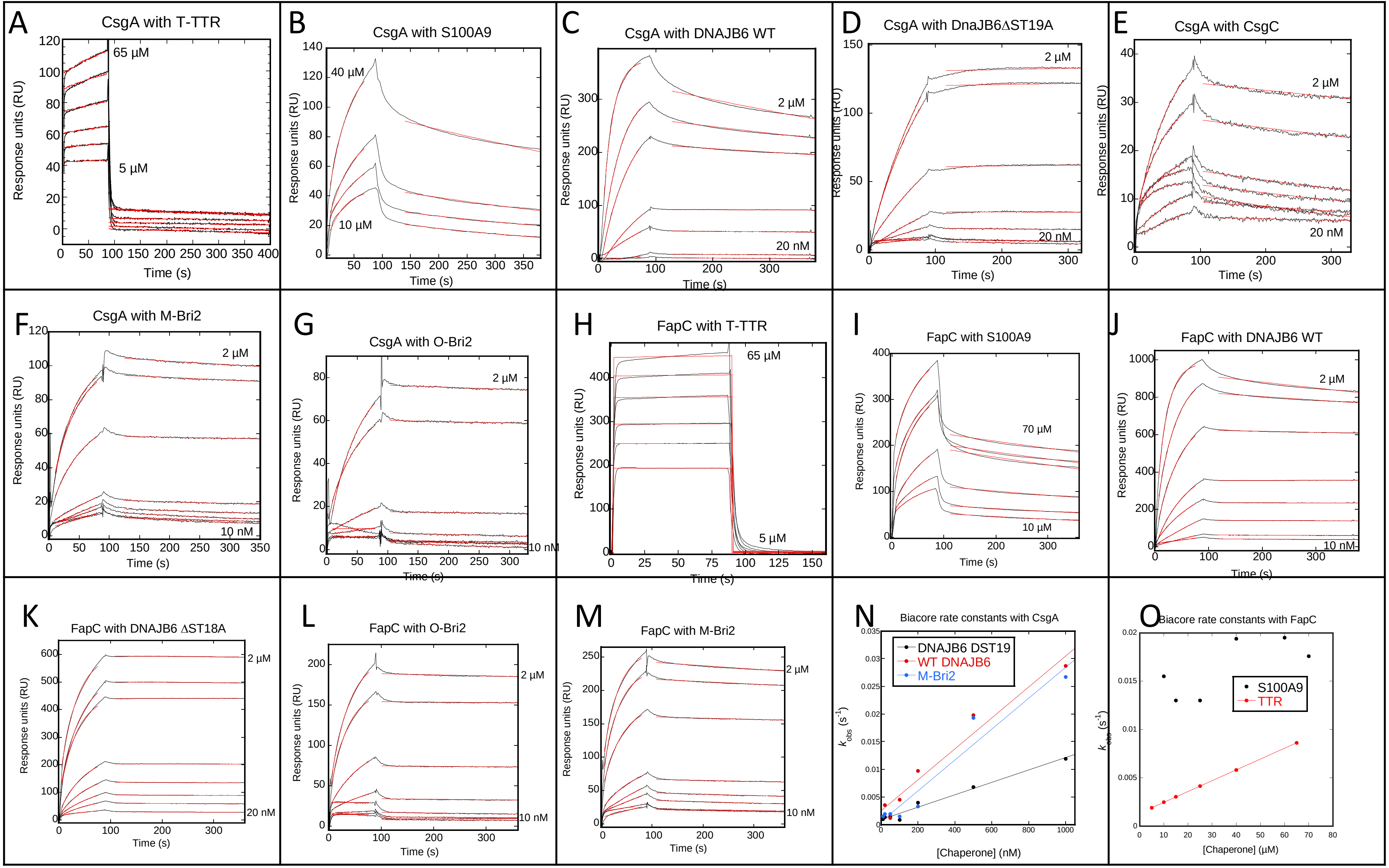

Figure S8. Data for binding of chaperones to immobilized CsgA or FapC by Biacore. Time curves shown in panels A-M with chaperone concentration ranges indicated. CsgA interaction with T-TTR (A), S100A9 (B), DNAJB6 WT (C), DNAJB6 ΔST19A (D), CsgC (E), M-Bri2 (F), O-Bri2 (G). FapC interaction with T-TTR (H), S100A9 (I), DNAJB6 WT (J), DNAJB6 ΔST19A (K), O-Bri2 (L), M-Bri2 (M). Best fits to the binding region of the time curves (using a 1:1 binding model) are provided in red. Rate constants of association plotted versus chaperone concentration for CsgA (N) and FapC (O).

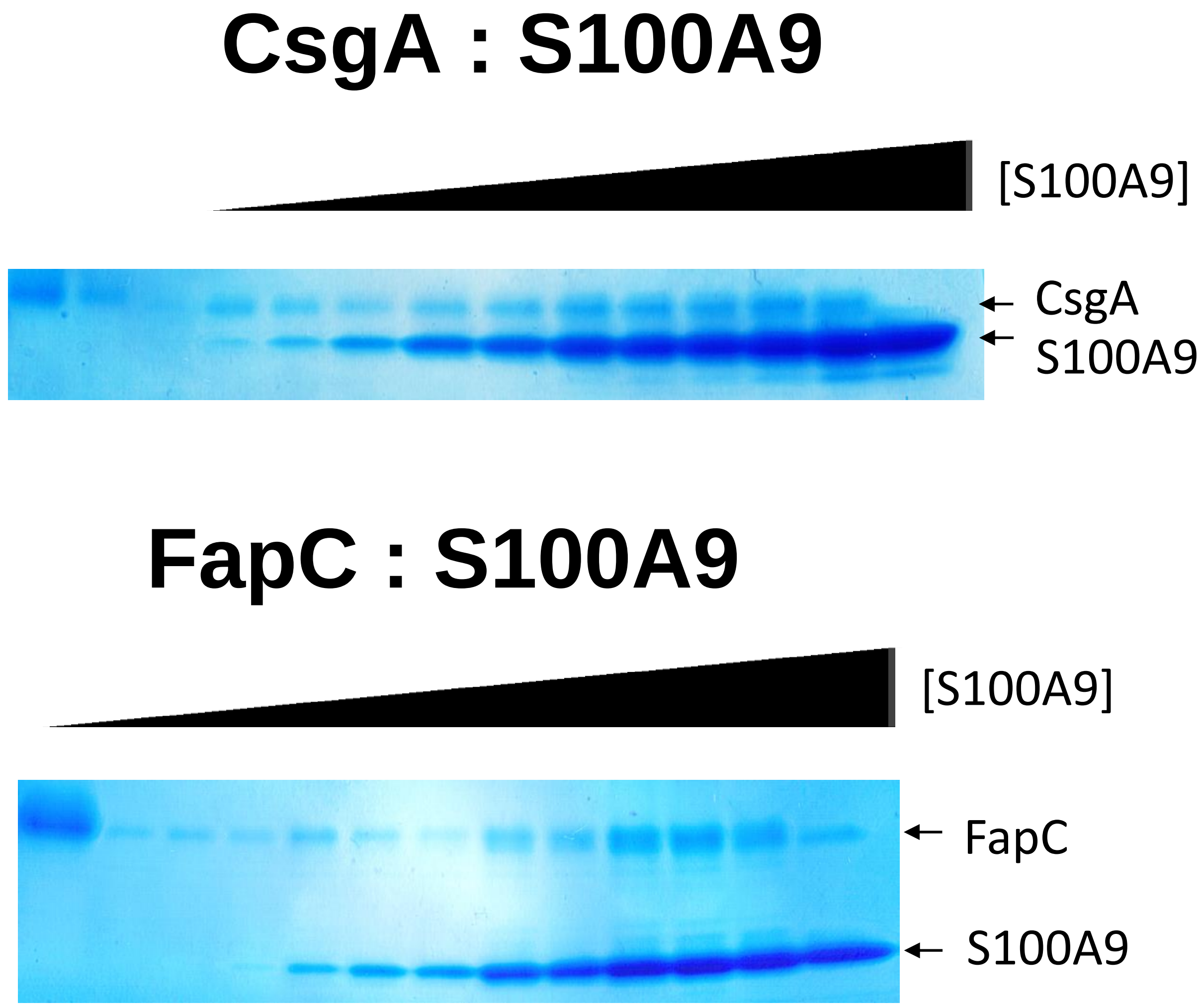

Figure S9. SDS-PAGE gel scans showing increasing solubility of CsgA and FapC when aggregated in the presence of increasing amounts of S100A9. After aggregation of CsgA or FapC in the presence of S100A9, the solution was spun down and the supernatant run on SDS-PAGE. Band intensities are shown in Fig. 5C.

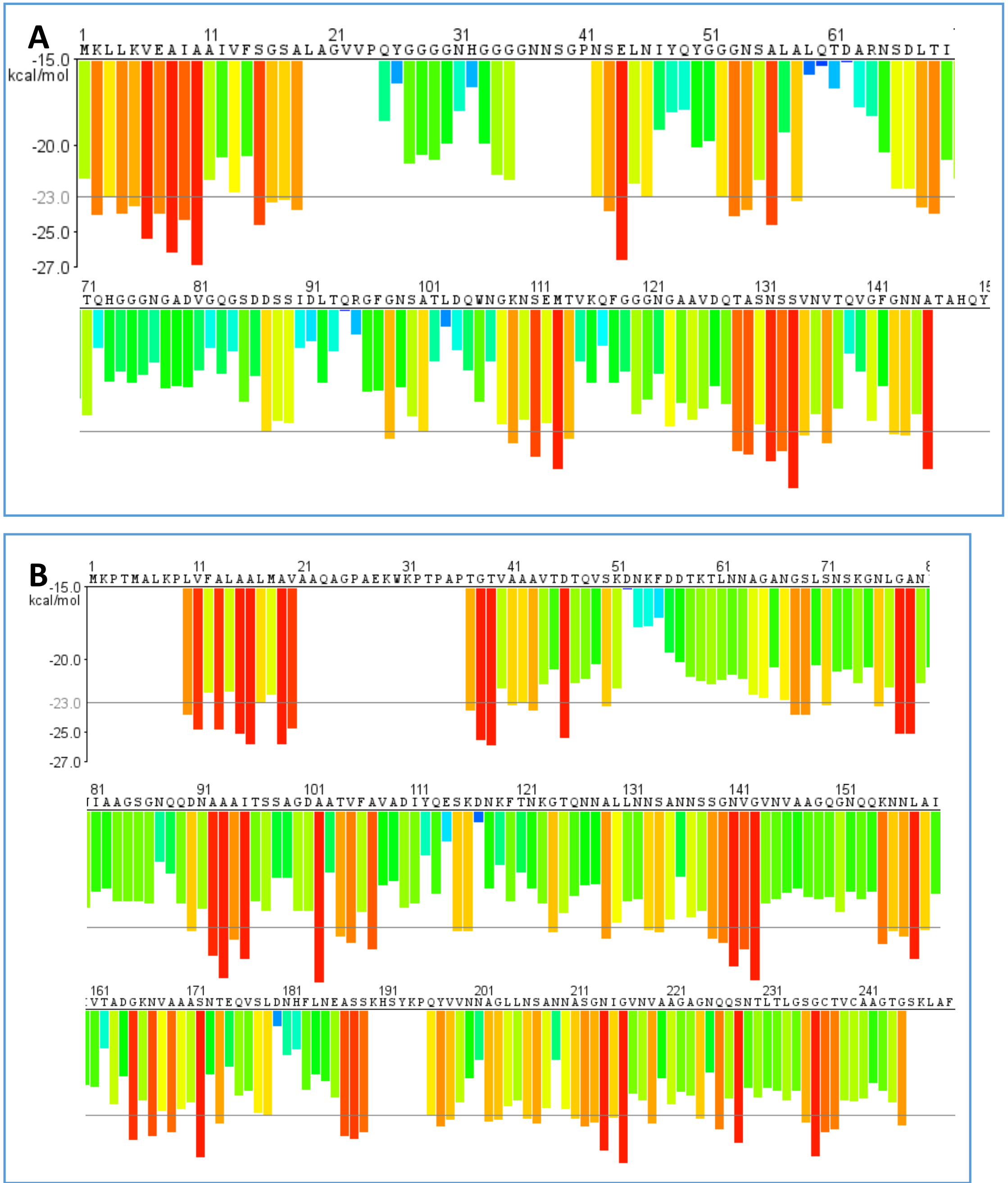

Figure S10. Computational prediction of aggregation propensity of A) CsgA and B) FapC sequences based on Rosetta energies that predict amyloid propensities of hexapeptide fragments within the sequence. Orange – red segments with energies below -23 kcal/mol are predicted to take part in fibril formation. The letters on the x-axis display the first residue of hexapeptides forming part of the CsgA or FapC sequence.
